# Pan-Cancer Integrated Analysis of HSF2 Expression, Prognostic Value and Potential Implications for Cancer Immunity

**DOI:** 10.3389/fmolb.2021.789703

**Published:** 2022-01-11

**Authors:** Fei Chen, Yumei Fan, Xiaopeng Liu, Jianhua Zhang, Yanan Shang, Bo Zhang, Bing Liu, Jiajie Hou, Pengxiu Cao, Ke Tan

**Affiliations:** ^1^ Ministry of Education Key Laboratory of Molecular and Cellular Biology, Key Laboratory of Animal Physiology, Biochemistry and Molecular Biology of Hebei Province, College of Life Sciences, Hebei Normal University, Shijiazhuang, China; ^2^ Department of Neurosurgery, The Second Hospital of Hebei Medical University, Shijiazhuang, China

**Keywords:** HSF2, pan-cancer, prognosis, immune infiltration, immune checkpoint genes, multi-omics

## Abstract

Heat shock factor 2 (HSF2), a transcription factor, plays significant roles in corticogenesis and spermatogenesis by regulating various target genes and signaling pathways. However, its expression, clinical significance and correlation with tumor-infiltrating immune cells across cancers have rarely been explored. In the present study, we comprehensively investigated the expression dysregulation and prognostic significance of HSF2, and the relationship with clinicopathological parameters and immune infiltration across cancers. The mRNA expression status of HSF2 was analyzed by TCGA, GTEx, and CCLE. Kaplan-Meier analysis and Cox regression were applied to explore the prognostic significance of HSF2 in different cancers. The relationship between HSF2 expression and DNA methylation, immune infiltration of different immune cells, immune checkpoints, tumor mutation burden (TMB), and microsatellite instability (MSI) were analyzed using data directly from the TCGA database. HSF2 expression was dysregulated in the human pan-cancer dataset. High expression of HSF2 was associated with poor overall survival (OS) in BRCA, KIRP, LIHC, and MESO but correlated with favorable OS in LAML, KIRC, and PAAD. The results of Cox regression and nomogram analyses revealed that HSF2 was an independent factor for KIRP, ACC, and LIHC prognosis. GO, KEGG, and GSEA results indicated that HSF2 was involved in various oncogenesis- and immunity-related signaling pathways. HSF2 expression was associated with TMB in 9 cancer types and associated with MSI in 5 cancer types, while there was a correlation between HSF2 expression and DNA methylation in 27 types of cancer. Additionally, HSF2 expression was correlated with immune cell infiltration, immune checkpoint genes, and the tumor immune microenvironment in various cancers, indicating that HSF2 could be a potential therapeutic target for immunotherapy. Our findings revealed the important roles of HSF2 across different cancer types.

## Introduction

The incidence and mortality of cancer are increasing rapidly every year worldwide, posing a serious threat to public health (Sung et al., 2021). Among the most common cancers, breast, lung, and liver are the main causes of high mortality worldwide. Although scientists have made considerable efforts to improve the diagnosis and treatment of cancer, the 5-years survival rate for cancer patients remains disappointing (Ferlay et al., 2021; Sung et al., 2021). Concurrently, the economic burden of cancer on countries worldwide is gradually increasing (Ferlay et al., 2021; Sung et al., 2021). Therefore, there is an urgent need to find diagnostic biomarkers and new treatments for cancer.

Cancer cells face multiple internal and external stresses that are distinct from those faced by normal cells (Jeggo et al., 2016). These stimuli cause dysfunction of proteostasis as a result of protein misfolding, gene mutation, oncogene activation, inhibition of tumor suppressors, chromosomal rearrangement, oxidative stress, hypoxia, and impaired degradation of proteins (Hanahan and Weinberg, 2011; Jeggo et al., 2016). Upon exposure to these various stimulators, heat shock factor (HSF), which is the original regulator of the heat shock response (HSR), controls the rapid and dynamic expression of heat shock proteins (HSPs) (Akerfelt et al., 2010; Fujimoto and Nakai, 2010; Gomez-Pastor et al., 2018). HSPs, acting as molecular chaperones, are involved in various physiological and pathological processes, such as the folding and assembly of nascent polypeptides and the intracellular transport of proteins, and to exhibit cytoprotective effects. The HSF family contains five members, including HSF1, HSF2, HSF4, HSF5, and HSFY (Akerfelt et al., 2010; Fujimoto and Nakai, 2010; Gomez-Pastor et al., 2018). Numerous studies have demonstrated that HSF1 is associated with DNA damage repair, reprogrammed metabolism oncogenesis, and metastasis (Mendillo et al., 2012; Dai and Sampson, 2016; Dai, 2018; Wang G. et al., 2020; Puustinen and Sistonen, 2020). Thus, HSF1 is believed to be a potential therapeutic target for anticancer therapy (Zhang B. et al., 2021; Chen et al., 2021).

In contrast to HSF1, HSF2 has been shown to play an important role in mediating organ development, differentiation, and the ubiquitin proteasome pathway (Rallu et al., 1997; Mathew et al., 1998; Widlak and Vydra, 2017). HSF2 is necessary for embryogenesis and spermatogenesis as evidenced by knocking out the *HSF2* gene in mice (Sarge et al., 1994; Rallu et al., 1997; Björk et al., 2010). Apoptosis of spermatocytes is remarkably increased, and the maturation of male germ cells is impaired in *HSF2*-null mice (Sarge et al., 1994; Rallu et al., 1997; Björk et al., 2010). A recent study revealed that HSF2 promoted spermatogenesis by regulating the expression of HSP and Y chromosomal multicopy genes, including SLX, SLY, and SSTY2 (Akerfelt et al., 2008). HSF2 is also associated with brain development, as evidenced by HSF2-null mice exhibiting enlarged ventricles, a small hippocampus, and neurons mispositioning (Kallio et al., 2002; Wang et al., 2003; Chang et al., 2006). As a member of the HSF family, previous studies have suggested that HSF2 could form heterotrimers with HSF1 to promote the transcription of HSP and some other genes (Sistonen et al., 1994; Ostling et al., 2007; Sandqvist et al., 2009). However, the precise function and molecular mechanisms of HSF2 in tumorigenesis still need to be explored.

Although increasing evidence indicates that HSF2 may play a vital role in the tumorigenesis of some specific types of cancers, a systematic pan-cancer analysis of HSF2 has not yet been conducted. Therefore, the aims of this study were to explore the expression profile, prognostic value, methylation level of HSF2, and potential relationship between HSF2 expression and immunological functions in 33 different types of cancer.

## Materials and Methods

### Heat Shock Factor 2 Expression in Pan-Cancer

The Cancer Genome Atlas (TCGA, https://www.cancer.gov/) database, which is widely used for comprehensive analyses of human cancers, was employed to investigate the differential expression of HSF2 across different cancer types. RNA sequencing data and clinical follow-up information for patients with 33 types of cancers were downloaded from the TCGA database. Because the normal tissues sequencing data included in the TCGA are very limited and many patients lack transcriptome sequencing results for their normal tissues, we obtained data for normal tissues from the Genotype-Tissue Expression (GTEx) database. The cell line expression matrix of HSF2 in pan-cancer was obtained from the CCLE dataset (https://portals.broadinstitute.org/ccle/about). The above analyses were constructed using the R (v4.0.3) software package ggplot2 (v3.3.3). R software v4.0.3 and ggplot2 (v3.3.3) were used for visualization. R software v4.0.3 was used for statistical analysis.

### Heat Shock Factor 2 Expression and its Clinical Correlation in Pan-Cancer

The correlations of HSF2 expression with tumor stage and DNA methylation were investigated using the UALCAN database (http://ualcan.path.uab.edu/).

### Gene Ontology (GO), Kyoto Encyclopedia of Genes and Genomes (KEGG), and Gene Set Enrichment Analysis (GSEA)

GO, KEGG, and GSEA were conducted to examine the biological and molecular functions of HSF2 across different cancer types using a total of 300 genes that were positively correlated with HSF2. GO analysis was applied to investigate the BP, CC, and MF associated with HSF2 in different cancers. All three analyses were performed using the R package Cluster Profiler.

### cBioPortal Database

The genetic alterations of HSF2 in different cancer types were obtained using the cBioPortal database.

### The Prognostic Potential of Heat Shock Factor 2 in Pan-Cancer

The survival data from 33 types of cancer were obtained from the TCGA database for further overall survival (OS), disease-specific survival (DSS), disease-free interval (DFI), and progression-free interval (PFI) analyses. Univariate Cox regression analysis was used to analyze HSF2-related survival with the R package limma, survival, and forestplot to show the *p* value, HR, and 95% CI. The Kaplan-Meier (KM) method was used to investigate the prognostic value of HSF2 in human cancers using the R packages limma, survival, and survminer. R software v4.0.3 was used for statistical analysis.

### Univariate and Multivariate Cox Regression Analyses and Construction of a Nomogram

Cox regression analysis, including univariate, and multivariate analyses, was used to examine the prognostic value of HSF2 in KIRP, ACC, and LIHC. The forest plot was constructed using the R package “forest plot” to exhibit the hazard ratio (HR), 95% CI, and *p*-value. The nomogram was constructed using the R package “rms”.

### Correlation of Heat Shock Factor 2 Expression With Tumor Cell Infiltration and Immune Modulator Genes in Pan-Cancer

We obtained the data for 33 types of human cancer in TCGA from the GDC data portal website. For reliable immune score evaluation, we used the R software package “Immuneeconv” to integrate the two latest algorithms, including TIMER, and xCell. Heatmaps of the immune infiltration scores or immune modulator genes and HSF2 expression in different cancer types were generated with Spearman correlation analysis. The horizontal axis in the heatmaps shows the type of cancer, the vertical axis shows different immune cell infiltration scores, and the color shows the correlation coefficients. Additionally, R software v4.0.3 was used for statistical analysis.

### Relationships Between Heat Shock Factor 2 Expression and TMB or MSI in Pan-Cancer

We obtained the data for 33 types of human cancer in TCGA from the GDC data portal website. For pan-cancer analysis, the horizontal axis shows the correlation coefficient between HSF2 expression and TMB/MSI, the ordinate is the type of cancer, the size of the dots in the figure shows the degree of the correlation coefficient, and the different colors represent the significance of the *p* value. Correlation analysis between HSF2 and TMB/MSI was performed using Spearman’s method and R software v4.0.3 was used for statistical analysis. A *p*-value less than 0.05 was considered statistically significant.

## Results

### Heat Shock Factor 2 is Abnormally Expressed in Human Pan-Cancer

**FIGURE 1:**
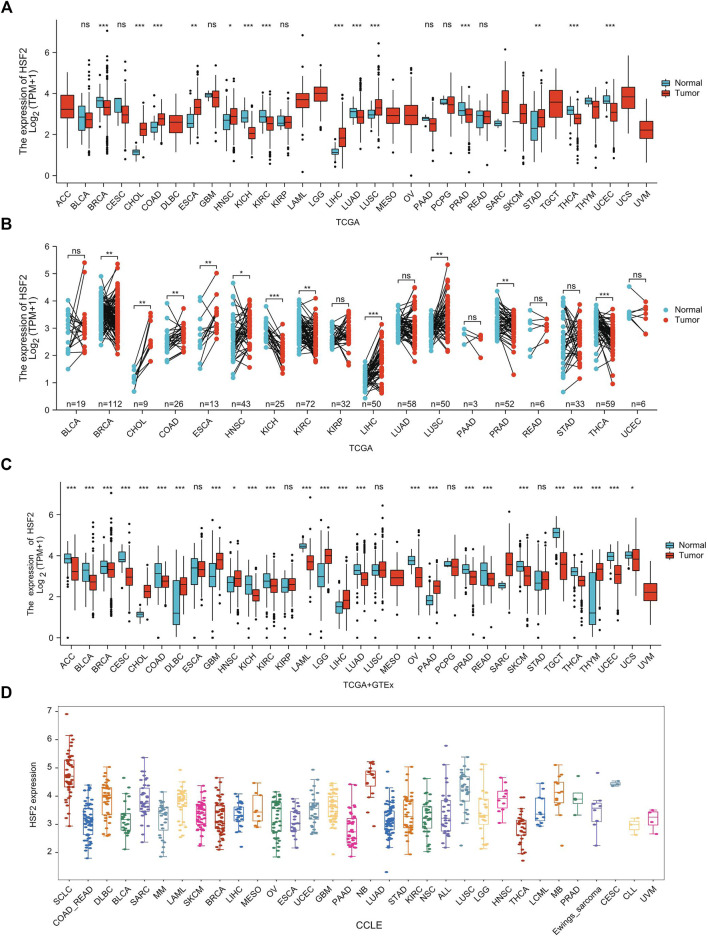
HSF2 expression levels in pan-cancer. **(A)** Upregulated or downregulated expression of HSF2 in various human cancers from TCGA datasets. **(B)** Increased or decreased expression of HSF2 in paired cancer tissues and adjacent normal tissues from TCGA datasets. **(C)** HSF2 differential expression across different cancer types in the TCGA and GTEx databases. **(D)** The mRNA level of HSF2 in different cancer cells according to the CCLE database. *p < 0.05; **p < 0.01; ***p < 0.001.

Based on the results from The Cancer Genome Atlas (TCGA) data alone, HSF2 expression was increased in CHOL, COAD, ESCA, HNSC, LIHC, LUSC, and STAD, but decreased in BRCA, KICH, KIRC, LUAD, PRAD, THCA, and UCEC tissues compared with adjacent normal tissues (Figure 1A). We also estimated HSF2 expression in paired cancer tissues and adjacent normal tissues in pan-cancer using TCGA datasets. HSF2 expression was significantly higher in CHOL, COAD, ESCA, HNSC, LIHC, and LUSC, but remarkably lower in BRCA, KICH, KIRC, PRAD, and THCA than in paired adjacent normal tissues (Figure 1B). Because several cancers lack corresponding normal tissue controls, we therefore combined the data from the TCGA, and Genotype Tissue-Expression (GTEx) (Figure 1C). After combining the data from TCGA and GTEx, the expression difference of HSF2 achieved significance in 25 out of 33 cancer types. HSF2 expression was higher in CHOL, DLBC, GBM, HNSC, LGG, LIHC, PAAD, and THYM but lower in ACC, BLCA, BRCA, CESC, COAD, KICH, KIRC, LAML, LUAD, OV, PRAD, READ, SKCM, TGCT, THCA, UCEC, and UCS (Figure 1C). Moreover, we also investigated the expression of HSF2 in different cancer cell lines according to the Cancer Cell Line Encyclopedia (CCLE) database (Figure 1D).

### Association of Heat Shock Factor 2 Expression With Clinicopathological Features in Different Cancer Types

**FIGURE 2:**
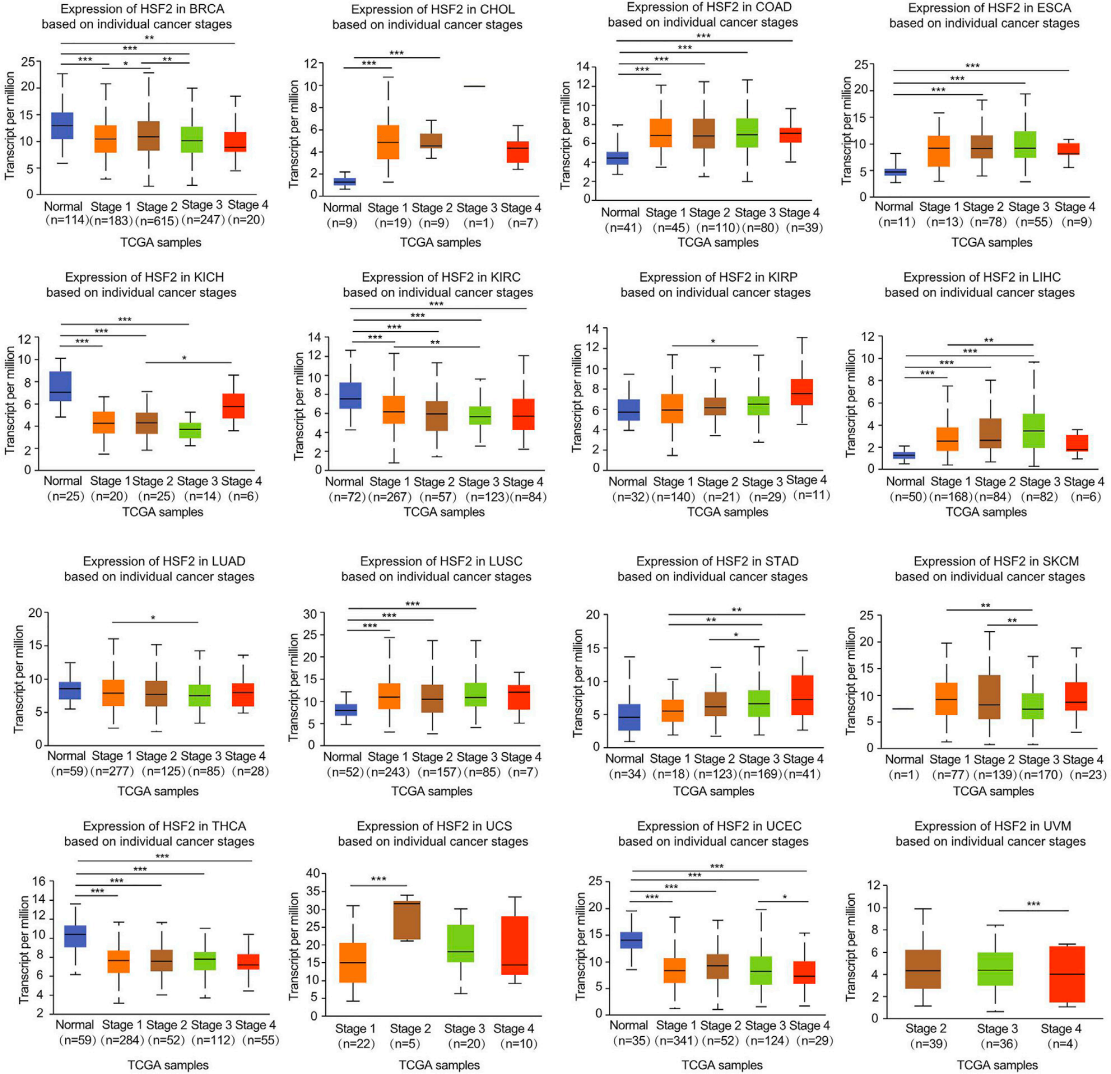
Correlation of HSF2 expression and clinicopathological parameters across different cancer types. The clinical correlations between HSF2 expression levels and tumor stage in different cancer types were examined using the UALCAN database. *p < 0.05; **p < 0.01; ***p < 0.001.

The relationship between HSF2 expression and the clinicopathological characteristics of patients with different cancers was investigated based on individual cancer stages, including stages 1, 2, 3, and 4. HSF2 expression was generally increased in CHOL, COAD, ESCA, KIRP, LIHC, LUSC, STAD, and UCS (Figure 2). In contrast, HSF2 expression was dramatically decreased in BRCA, KIRC, KICH, LUAD, SKCM, THCA, UCEC, and UVM (Figure 2). Moreover, HSF2 expression was stable in some cancers, including ACC, BLCA, CESC, DLBC, HNSC, MESO, OV, PAAD, READ, and TGCT (Supplementary Figure S1).

### Prognostic Values of Heat Shock Factor 2 in Human Pan-Cancer

**FIGURE 3:**
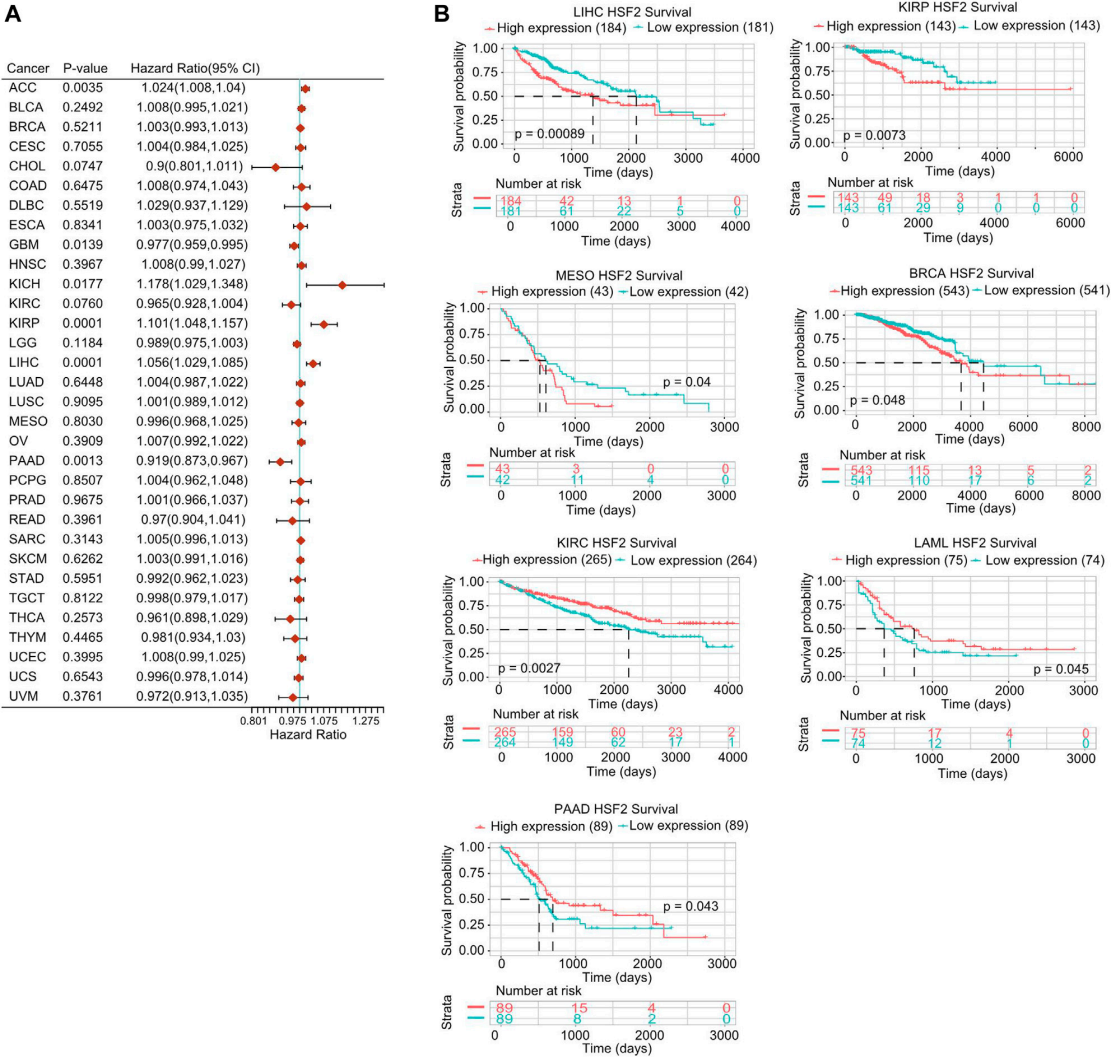
Prognostic potential of HSF2 in pan-cancer. **(A)** Correlation analysis of HSF2 expression with OS by the Cox regression model in various cancers. **(B)** OS curves comparing high and low expression of HSF2 in multiple cancer types using Kaplan-Meier methodology.

Next, we investigated the interrelationship between HSF2 expression and the prognosis of pan-cancer patients, including overall survival (OS), disease-specific survival (DSS), disease-free interval (DFI), and progression-free interval (PFI). Regarding the OS analysis, Cox regression results from 33 types of cancer suggested that HSF2 expression was markedly related to OS in 6 types of cancer, including ACC, GBM, KICH, KIRP, LIHC, and PAAD (Figure 3A). The results from the Kaplan-Meier (KM) survival curves demonstrated that higher HSF2 expression was correlated with worse OS in BRCA, LIHC, KIRP, and MESO, but with better OS in LAML, KIRC, and PAAD (Figure 3B). Moreover, we explored the relationship between HSF2 expression and DSS in cancer patients. As shown in Supplementary Figure S2A, HSF2 expression was associated with poor DSS in three types of cancer, including KIRP, LIHC, and UCEC. KM of DSS analysis indicated that upregulated HSF2 expression corresponded with poor DSS in patients with KIRP, LIHC, and KICH but with favorable DSS in patients with KIRC (Supplementary Figure S2B). Moreover, Cox regression analysis of PFI demonstrated that upregulated HSF2 expression was a risk factor in ACC, KICH, KIRP, and LIHC and was a protective factor in PAAD (Supplementary Figure S3A). Results from the KM of PFI analysis suggested that increased HSF2 expression was associated with a poor PFI in ACC and LIHC but with a favorable PFI in CHOL, KIRC, and LGG (Supplementary Figure S3B). Subsequently, we also assessed the association between HSF2 expression and DFI and identified that dysregulated HSF2 expression influenced DFI in patients with KIRP, LIHC, and UCEC (Supplementary Figure S4A). KM DFI analysis revealed that increased HSF2 mRNA expression was correlated with an unfavorable DFI in BLCA, CESC, and LIHC (Supplementary Figure S4B).

### Heat Shock Factor 2 is an Independent Prognostic Factor in KIPR, ACC, and LIHC

**FIGURE 4:**
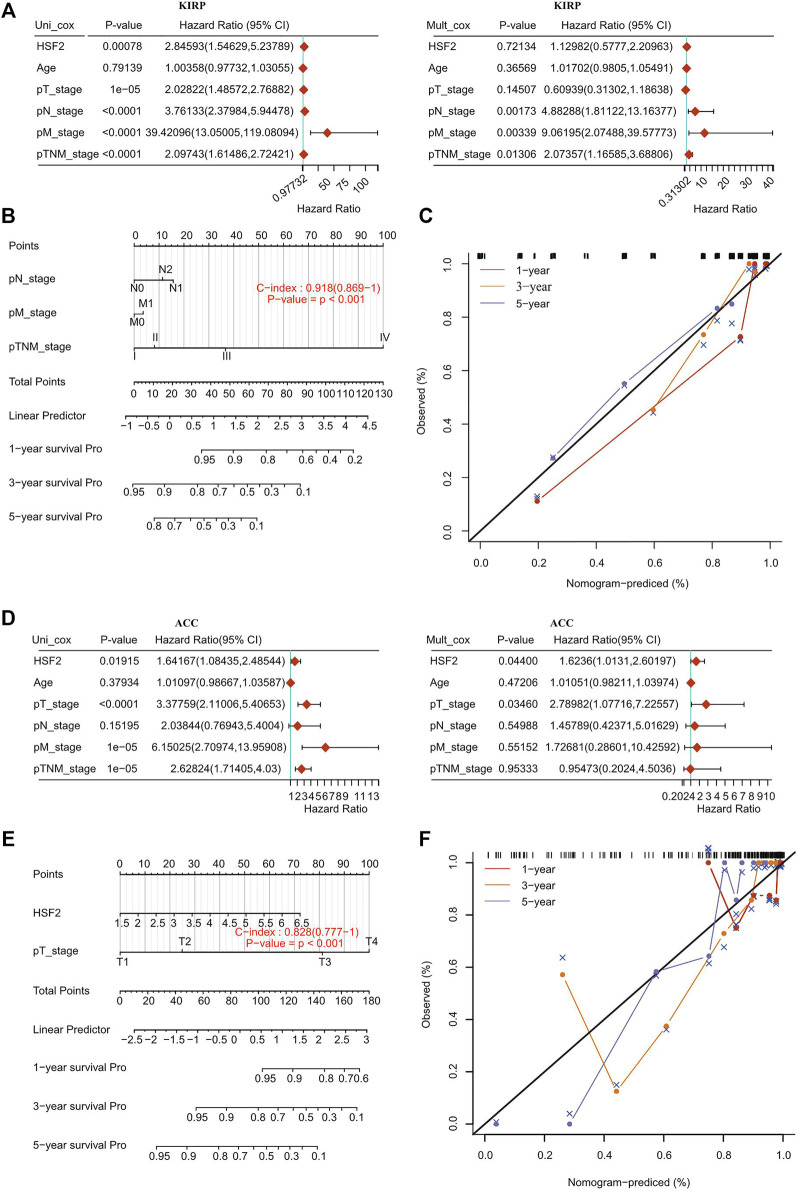
Internal validation of HSF2 as an independent prognostic factor for KIRP patients and ACC patients. **(A,D)** Univariate and multivariate Cox regression analyses were performed to determine HSF2 as an independent prognostic factor. **(B,E)** A prognostic nomogram integrating HSF2 expression and clinicopathologic variables was constructed to estimate OS. **(C,F)** Calibration plots to predict the OS of KIRP and ACC at 1, 3, and 5 years.

To further confirm whether HSF2 was an independent prognostic factor in cancers, univariate and multivariate Cox regression analyses were performed based on various clinicopathological characteristics, such as age, T stage, N stage, M stage, TNM stage, and grade. Univariate Cox regression analysis demonstrated that HSF2 expression (*p* < 0.001), T stage (*p* < 0.001), N stage (*p* < 0.001), M stage (*p* < 0.001), and TNM stage (*p* < 0.001) were significantly correlated with OS in KIPR (Figure 4A); HSF2 expression (*p* < 0.05), T stage (*p* < 0.001), M stage (*p* < 0.001), and TNM stage (*p* < 0.001) were obviously correlated with OS in ACC (Figure 4D); HSF2 expression (*p* < 0.001), M stage (*p* < 0.05) and TNM stage (*p* < 0.001) were strongly correlated with OS in LIHC (Supplementary Figure S5A). Multivariate analysis indicated that N stage (*p* < 0.01), M stage (*p* < 0.01), and TNM stage (*p* < 0.05) were significantly correlated with OS in KIPR (Figure 4A); HSF2 expression (*p* < 0.05) and T stage (*p* < 0.05) were obviously correlated with OS in ACC (Figure 4D); HSF2 expression (*p* < 0.001) and TNM stage (*p* < 0.001) were markedly correlated with OS in LIHC (Supplementary Figure S5A). In addition, a nomogram was constructed based on multivariate analysis (Figures 4B,E; Supplementary Figure S5B). The C-index and calibration curve confirmed the accuracy in predicting the 1-, 3-, and 5-years survival rates of cancer patients. The C-index of the prognostic nomogram was 0.918, 0.828, and 0.696 in KIPR, ACC, and LIHC, respectively (Figures 4C,F; Supplementary Figure S5C).

### DNA Methylation and Genetic Alteration Analysis of Heat Shock Factor 2 in Pan-Cancer

**FIGURE 5:**
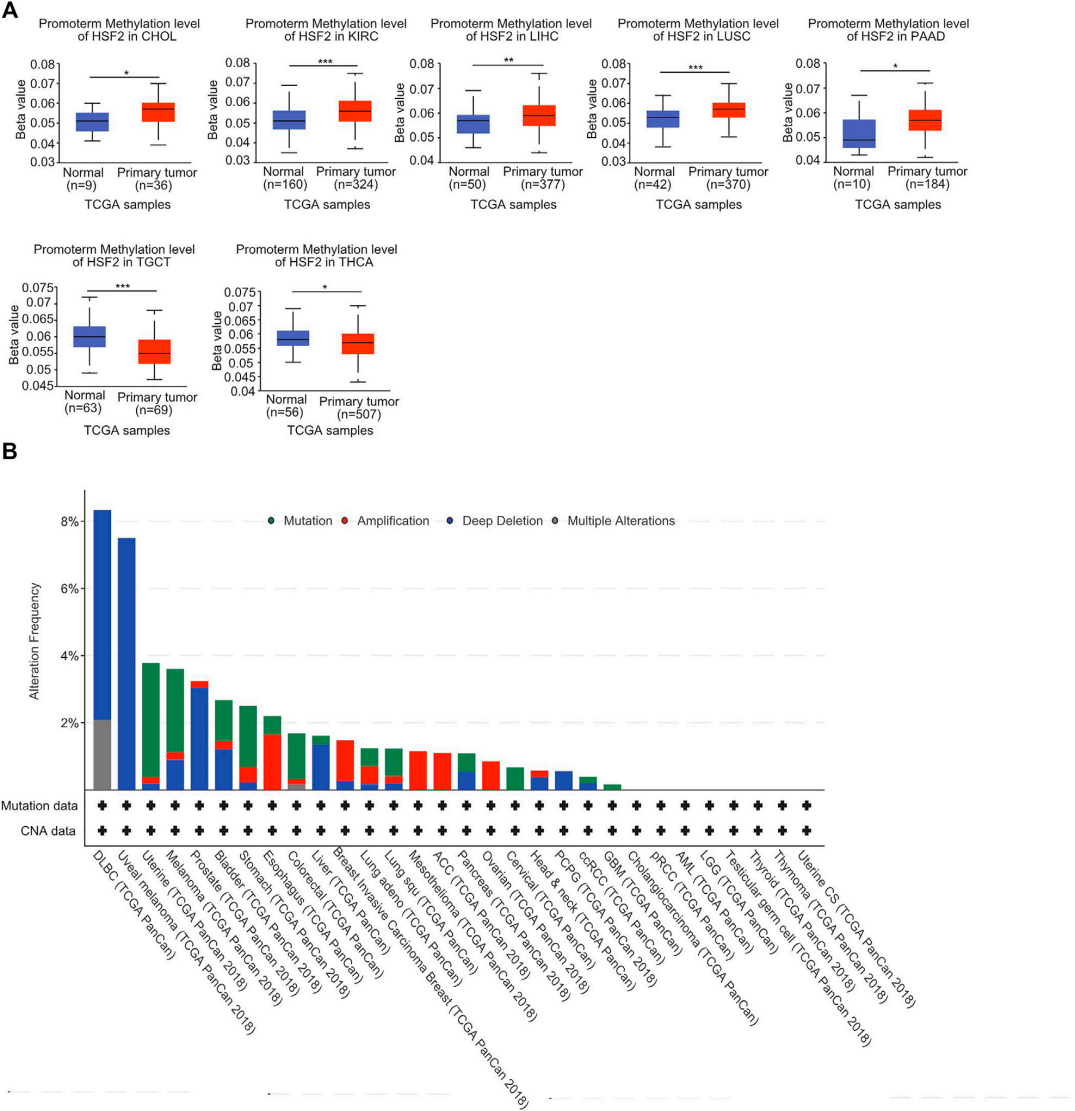
DNA methylation and mutation profile of HSF2 in pan-cancer. **(A)** The promoter methylation level of HSF2 in across different cancer types was investigated according to the UALCAN database. **(B)** The alteration frequency of HSF2 with different mutation types was obtained from the cBioPortal database. *p < 0.05; **p < 0.01; ***p < 0.001.

A growing body of evidence suggests that DNA methylation is an epigenetic molecular mechanism for gene expression and that DNA hypermethylation leads to the inactivation of a broad range of tumor suppressor genes. Therefore, we investigated the potential link between DNA methylation and HSF2 expression. With respect to the TCGA database, we observed that the DNA methylation level of HSF2 was obviously increased in CHOL, KIRC, LIHC, LUSC, and PAAD but decreased in TGCT and THCA based on the UALCAN database (Figure 5A). Moreover, we observed that DNA methylation was negatively correlated with HSF2 expression in many types of cancer, including ACC, BLCA, BRCA, CESC, CHOL, DLBC, ESCA, HNSC, KICH, KIRC, KIRP, LAML, LGG, LIHC, LUAD, LUSC, PAAD, PCPG, PRAD, SARC, SKCM, TGCT, THYM, UCEC, USC, and UVM (Supplementary Figure S6). In contrast, DNA methylation was positively associated with HSF2 expression in OV (Supplementary Figure S6).

In addition, we investigated the alteration frequency of HSF2 in different cancer types according to the cBioPortal database. The highest incidence rate of genetic variations of HSF2 was observed in DLBC, and deep depletion was the primary type (Figure 5B).

### GO and KEGG Analyses of Heat Shock Factor 2 in Pan-Cancer

**FIGURE 6:**
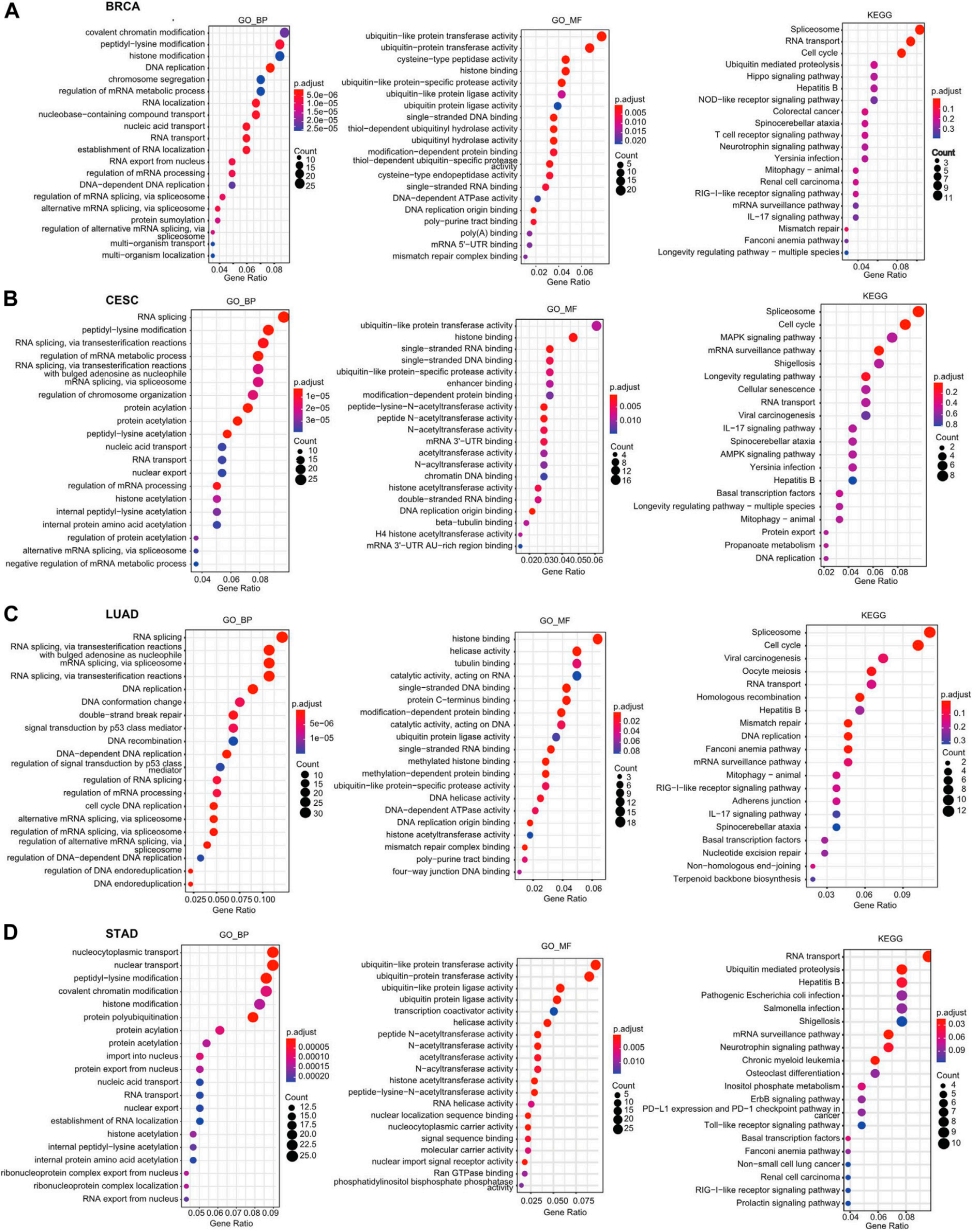
GO and KEGG enrichment analyses for HSF2 in cancers. Top 20 pathways enriched in the BP, MF, and KEGG analyses in **(A)** BRCA, **(B)** CESC, **(C)** LUAD, and **(D)** STAD.

First, we identified genes with positive or negative coexpression with HSF2 using the TCGA database (Supplementary Table S1), and the top 50 genes that were positively and negatively associated with HSF2 in different cancers are shown (Supplementary Figure S7). To explore the molecular mechanisms by which HSF2 regulates oncogenesis, we performed GO and KEGG analyses using the 300 genes that were positively related to HSF2 in several cancers (Figure 6). The top 5 enriched BP GO terms were covalent chromatin modification, peptidyl-lysine modification, histone modification, DNA replication, and chromosome segregation in BRCA; RNA splicing, peptidyl-lysine modification, regulation of mRNA metabolic process, regulation of chromosome organization, and protein acylation in CESC; RNA splicing, mRNA splicing, DNA replication, DNA conformation change, and double-strand break repair in LUAD; and nucleocytoplasmic transport, nuclear transport, peptidyl-lysine modification, covalent chromatin modification, and histone modification in STAD (Figures 6A–D). The top 3 enriched MF terms of GO were ubiquitin-like protein transferase activity, ubiquitin-protein transferase activity, and cysteine-type peptidase activity in BRCA; ubiquitin-like protein transferase activity, histone binding, and single-stranded DNA binding in CESC; ubiquitin-like protein transferase activity, ubiquitin-protein transferase activity, and ubiquitin-like protein ligase activity in LUAD; and histone binding, helicase activity, and tubulin binding in STAD (Figures 6A–D). The top 3 enriched CC terms of GO were nuclear speck, chromosomal region, and nuclear envelope in BRCA; nuclear chromatin, nuclear speck, and chromosomal region in CESC; chromosomal region, nuclear speck, and spindle in LUAD; and nuclear speck, nuclear envelope, and chromosomal region in STAD (Supplementary Figures S8A–D).

Moreover, KEGG pathway analysis suggested that HSF2 was associated with signaling pathways related to the spliceosome, RNA transport, cell cycle, ubiquitin-mediated proteolysis, and Hippo signaling pathway in BRCA; spliceosome, cell cycle, MAPK signaling pathway, mRNA surveillance pathway, and shigellosis in CESC; spliceosome, cell cycle, viral carcinogenesis, oocyte meiosis, and RNA transport in LUAD; and RNA transport, ubiquitin-mediated proteolysis, hepatitis B infection, pathogenic *Escherichia coli* infection, and Salmonella infection in STAD (Figures 6A–D).

### Heat Shock Factor 2-Related Signaling Pathways in Cancers Identified by GSEA

**FIGURE 7:**
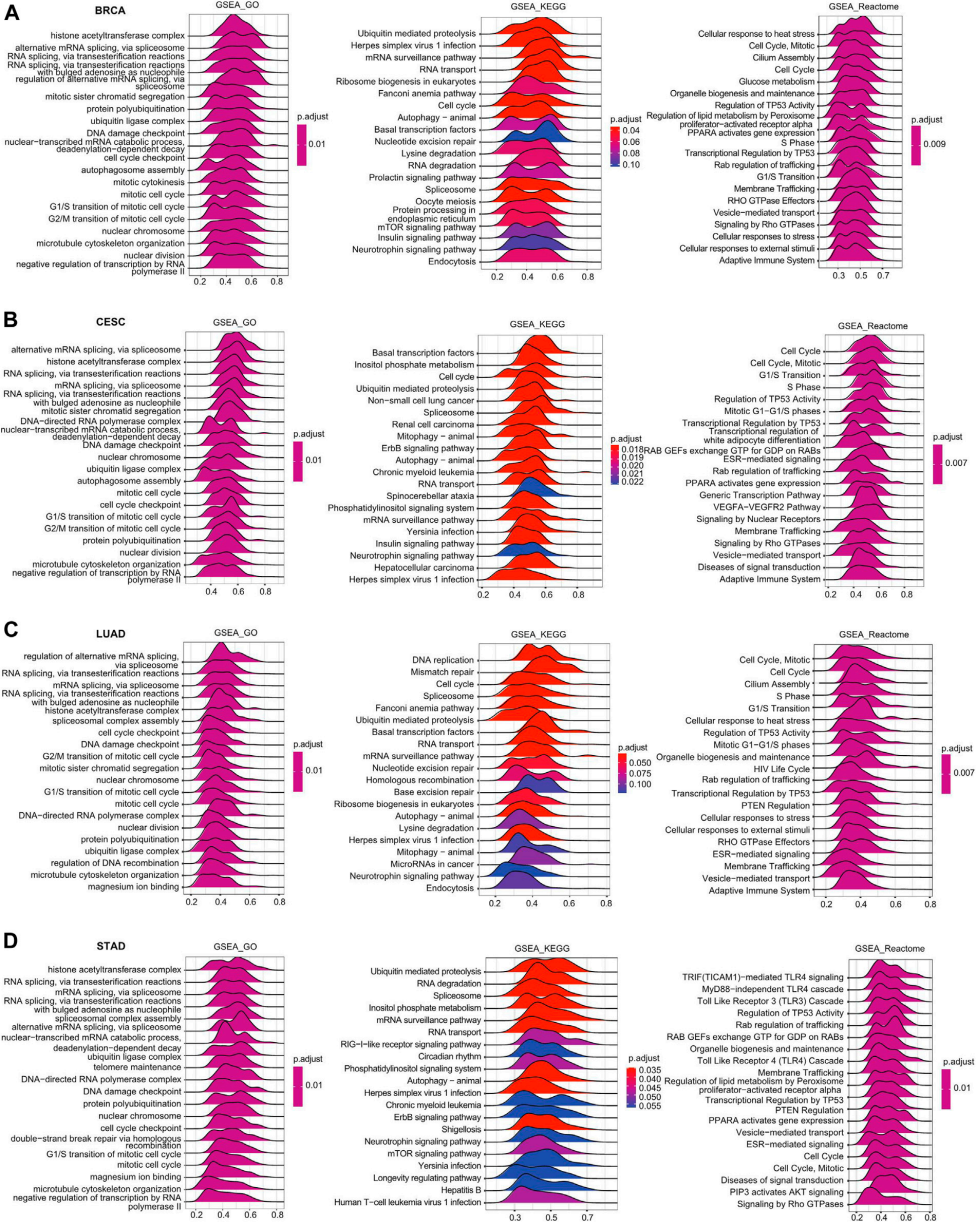
Merged enrichment plots for HSF2 according to GSEA in cancers. Merged plots of GSEA indicating the signaling pathways correlated with HSF2 based on the GO, KEGG, and Reactome analyses in **(A)** BRCA, **(B)** CESC, **(C)** LUAD, and **(D)** STAD.

GSEA was further performed to explore the signaling pathways and molecular mechanisms that were differentially affected by HSF2 in human cancers. Regarding the GO terms, the top 3 pathways influenced by HSF2 were the histone acetyltransferase complex, alternative RNA spicing via the spliceosome, and mitotic sister chromatid segregation in BRCA, CESC, LUAD, and STAD (Figures 7A–D). Among the KEGG terms, the top 3 pathways affected by HSF2 were ubiquitin-mediated proteolysis, herpes simplex virus 1 infection, and the mRNA surveillance pathway in BRCA; basal transcription factor, inositol phosphate metabolism, and cell cycle in CESC; DNA replication, mismatch repair and cell cycle in LUAD; and ubiquitin-mediated proteolysis, RNA degradation, and spliceosome in STAD (Figures 7A–D). More importantly, regarding the Reactome terms, the outcome of GSEA indicated that in addition to the cell response to stress, different immunity-related pathways were associated with HSF2, including the adaptive immune response, TRIF (TICAM1)-mediated TLR4 signaling, MyD88-dependent TLR4 cascade, TLR3 cascade, TLR4 cascade, and various bacterial or viral infections. Taken together, these findings imply that there is a close relationship among HSF2, the inflammatory response, and the tumor microenvironment (TME) (Figures 7A–D).

### Association of Heat Shock Factor 2 Expression and Immune Cell Infiltration in Pan-Cancer

**FIGURE 8:**
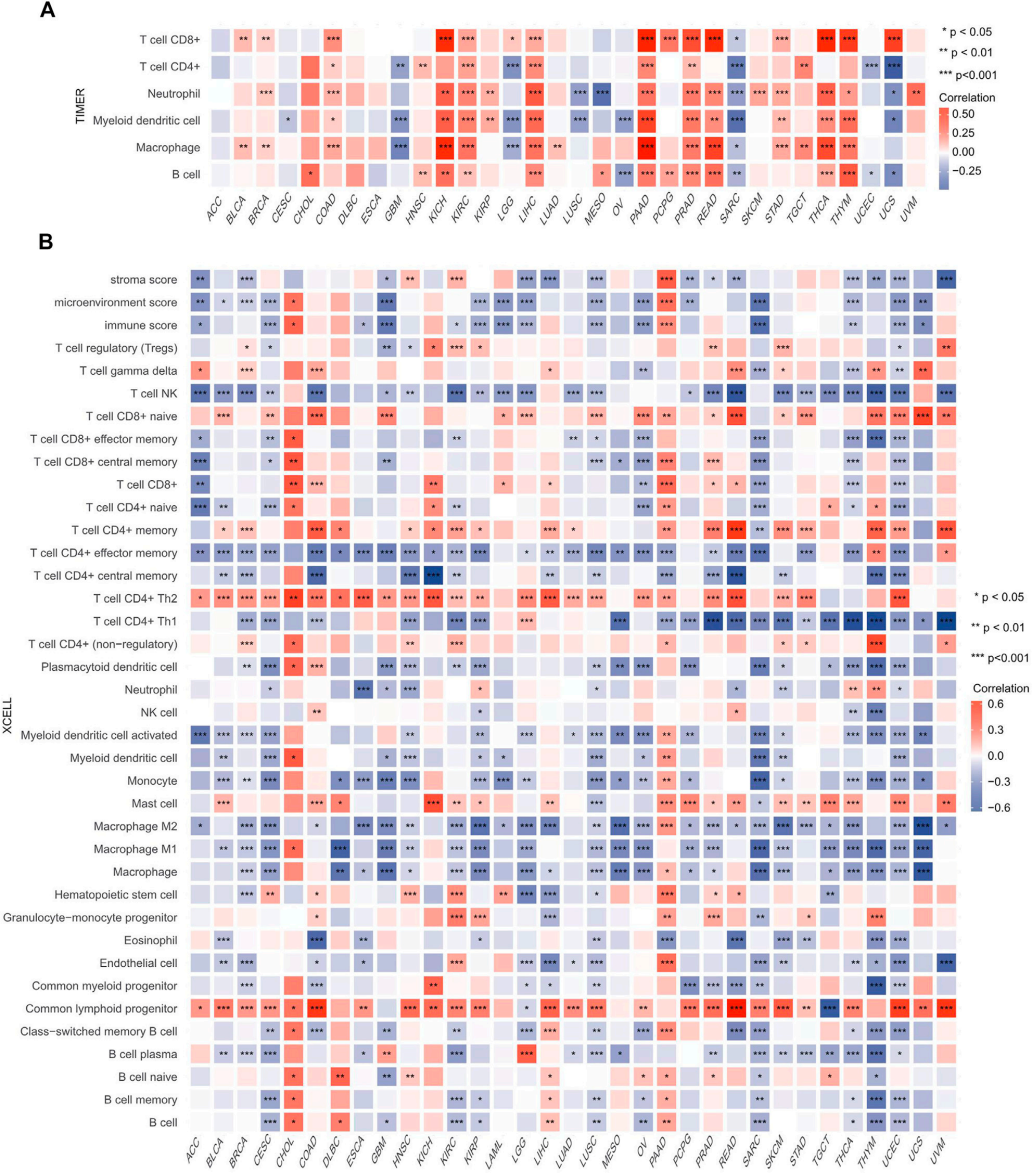
Correlations of HSF2 expression with the infiltration level of immune cells across different cancer types. **(A)** Heatmap of correlations between the expression of HSF2 and the level of immune infiltration in 32 types of human cancer using TIMER. **(B)** Heatmap of correlations between the expression of HSF2 and the level of immune infiltration in 33 types of human cancer using xCell. *p < 0.05; **p < 0.01; ***p < 0.001.

Because immune-infiltrating cells play an important role in cancer initiation and development, we then estimated the association between HSF2 expression and the infiltration levels of six major immune cells in 32 types of cancers. Using the data obtained from the TIMER database, the correlation between HSF2 expression, and the infiltration levels of these immune cells was investigated separately. The results implied that HSF2 expression was markedly correlated with the infiltrating level of B cells in 16 types of cancer, CD4^+^ T cells in 12 types of cancer, CD8^+^ T cells in 16 types of cancer, macrophages in 17 types of cancer, neutrophils in 18 types of cancer, and DCs in 18 types of cancer (Figure 8A). In addition, HSF2 positively correlated with these six types of immune cells in KIRC, LIHC, PAAD, and PRAD but negatively correlated with these immune cells in SARC (Figure 8A). To further confirm the relationship between HSF2 expression and infiltration of 38 subtypes of immune cell subtypes, we utilized the xCell database. HSF2 expression was negatively related to the infiltration levels of most immune cells in LUSC, SARC, and UCEC (Figure 8B).

### Relationships Between Heat Shock Factor 2 Expression and Immune Checkpoint Genes, Chemokines, Immunostimulators, and MHC-Related Genes in Pan-Cancer

**FIGURE 9:**
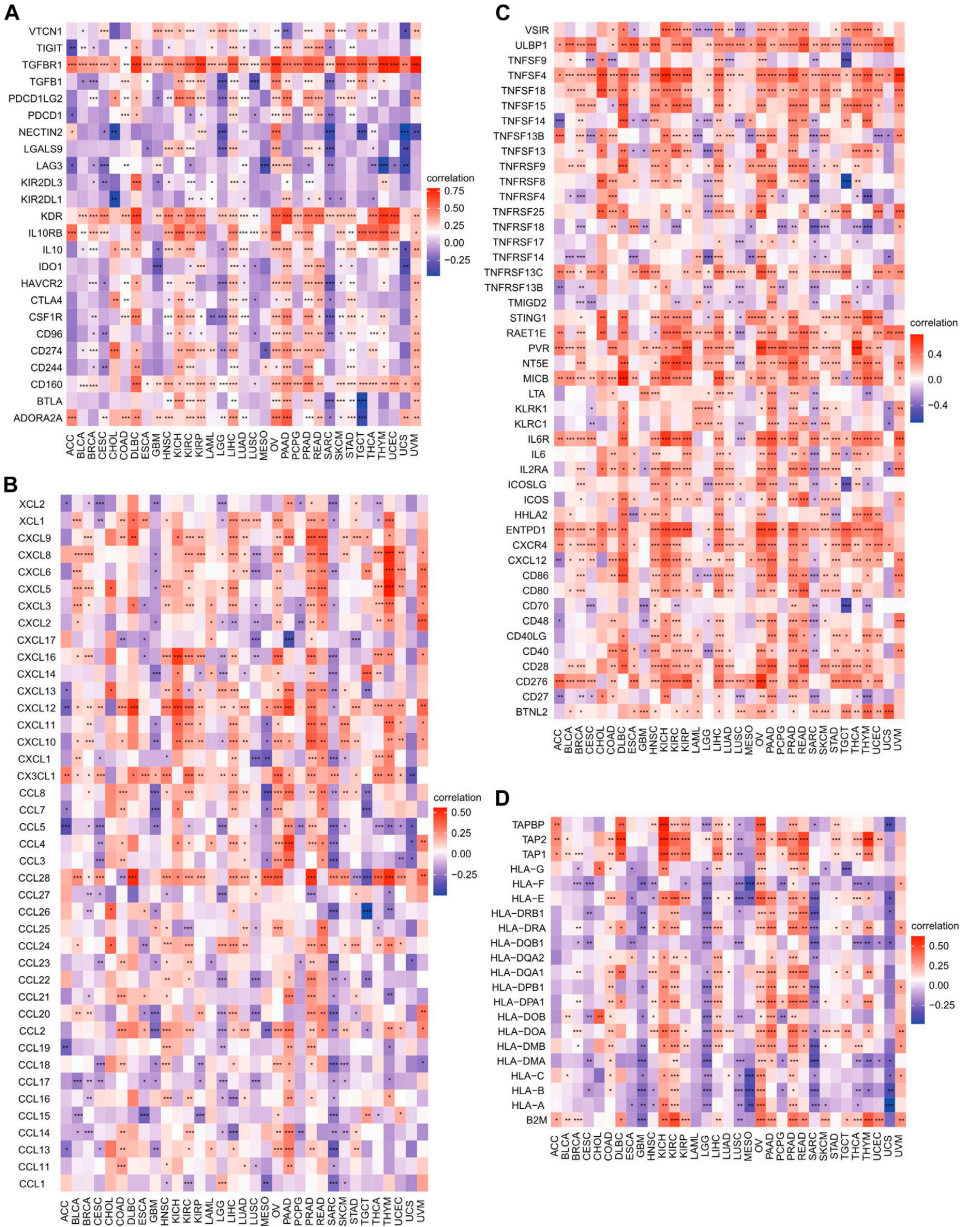
Correlations of HSF2 expression with immune checkpoint genes, chemokines, immunostimulators, and MHC-related genes across different cancer types. **(A)** Heatmap of correlations between HSF2 expression and immune checkpoint genes. **(B)** Heatmap of correlation between HSF2 expression and chemokines. **(C)** Heatmap of the correlation between HSF2 expression and immunostimulators. **(D)** Heatmap of the correlation between HSF2 expression and MHC-related genes. *p < 0.05; **p < 0.01; ***p < 0.001.

Because immune checkpoint genes play an important role in tumor immunotherapy, the correlations between HSF2 and immune checkpoint genes, immunoinhibitors, and immunostimulators were subsequently analyzed. Notably, we observed that HSF2 was significantly correlated with most immune checkpoint genes, including PD-1, PD-L1, CTLA4, KDR, TGFBR1, and IL10RB, in OV, PAAD, PRAD, and LIHC (Figure 9A). Interestingly, HSF2 expression was positively correlated with the expression of different chemokines in PAAD and PRAD but negatively associated with the expression of most chemokines in SRAC (Figure 9B). Moreover, we found that the expression of HSF2 was significantly and positively associated with immunostimulators in HNSC, KICH, KIRC, LIHC, OV, PAAD, and PRAD (Figure 9C). Additionally, HSF2 expression was positively correlated with most MHC-related genes in KIRC, OV, PAAD, and PRAD (Figure 9D). In contrast, HSF2 expression was negatively and strongly associated with most MHC-related genes in LGG and SARC (Figure 9D).

### Relationships Between Heat Shock Factor 2 Expression and TMB and MSI in Pan-Cancer

**FIGURE 10:**
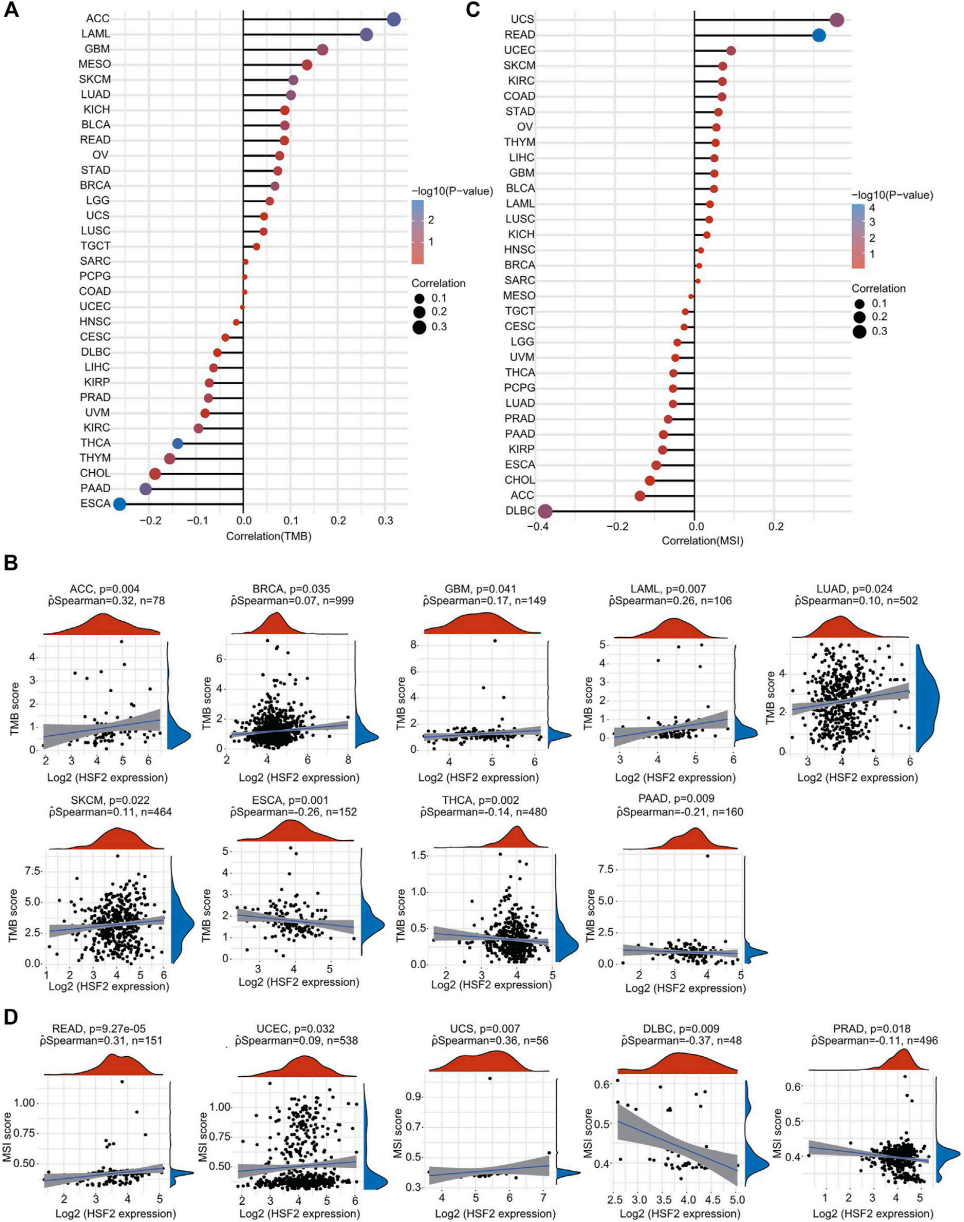
Correlations of HSF2 expression and TMB and MSI in pan-cancer. **(A)** The stick chart shows the associations between HSF2 expression and TMB in pan-cancer. **(B)** Relationship between HSF2 expression and TMB in 9 tumors types. **(C)** The stick chart shows the associations between HSF2 expression and MSI in pan-cancer. **(D)** Relationship between HSF2 expression and MSI in 5 tumors types. Correlation analysis was performed using Spearman’s method.

TMB and MSI are two emerging biomarkers associated with the immunotherapy response. The relationships between HSF2 expression level and TMB across different cancer types were also investigated. The expression level of HSF2 was markedly and positively correlated with TMB in many cancers, including ACC, BRCA, GBM, LAML, LUAD, and SKCM, but negatively correlated with TMB in ESCA, THCA, and PAAD (Figures 10A,B). Additionally, the correlation of HSF2 expression with MSI was also explored in pan-cancer, among which READ, UCEC, and UCS exhibited a positive correlation while DLBC and PRAD exhibited a negative correlation with HSF2 expression (Figures 10C,D).

### Effect of Heat Shock Factor 2 Expression on the Expression of Immune Checkpoints

**FIGURE 11:**
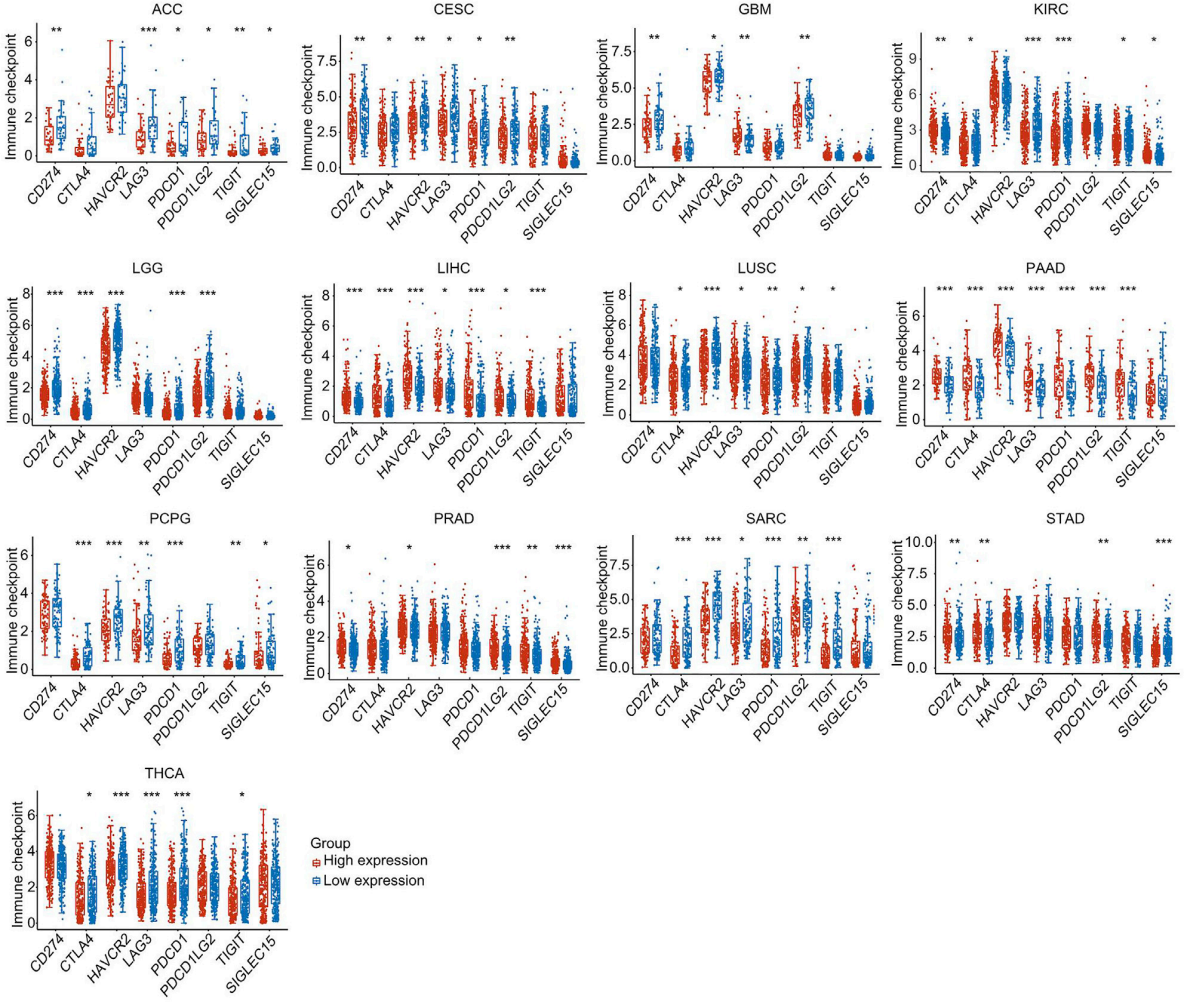
The expression of various immune checkpoint genes between the HSF2 low-expression group and the high-expression group in different cancer types. *p < 0.05, **p < 0.01, ***p < 0.001.

Cancer patients were separated into high-expression and low-expression groups based on HSF2 expression. We then evaluated the effect of HSF2 expression on the expression of well-known immune checkpoints (CD274, CTLA4, HAVCR2, LAG3, PDCD1, PDCD1LG2, TIGHT, and SIGLEC15) to estimate the immunotherapy responses correlated with HSF2 expression. Significantly lower expression of most immune checkpoint genes was observed in the HSF2 high-expression group than in the HSF2 low-expression group in ACC, CESC, GBM, LGG, LUSC, PCPG, SARC, and THCA (Figure 11). In contrast, the expression of most immune checkpoint genes was higher in the HSF2 high-expression than low-expression group in LIHC, PAAD, PRAD, and STAD (Figure 11).

## Discussion

Cancer has become a serious threat to human health worldwide due to its high morbidity and mortality (Ferlay et al., 2021; Sung et al., 2021). Early detection and effective treatment are important prerequisites for improving the prognosis of cancer patients (Zhong et al., 2016; Fan et al., 2021; Liu et al., 2021). At present, the most common cancer treatments include surgical resection, radiation, and adjuvant chemotherapy, but the effectiveness is still limited. Therefore, it is urgent and necessary to identify novel tumor biomarkers and to understand their molecular mechanisms involved in tumorigenesis and progression for the development of more effective diagnostic methods and treatment strategies. HSF2, a transcription factor for the heat shock response, plays significant roles in corticogenesis and spermatogenesis by regulating various target genes, and signaling pathways (Rallu et al., 1997; Mathew et al., 1998; Widlak and Vydra, 2017). Nevertheless, HSF2 has not been largely studied in the cancer field, and its role in oncogenesis or pan-cancer is still unclear. In the present study, we employed an array of bioinformatics methods to explore the potential tumor-promoting or tumor-suppressing roles of HSF2 by investigating the significant correlation between HSF2 expression and the prognosis of cancer patients, DNA methylation, TMB, MSI, immune cell infiltration levels, and immune checkpoint genes in pan-cancer according to the results from the TCGA, GTEx, UALCAN, and cBioPortal databases.

Here, we conducted the first comprehensive systematic analysis of HSF2 across 33 cancer types. Our results showed that HSF2 was dysregulated in various human cancers, which was consistent with previous studies from other clinical and preclinical data (Mustafa et al., 2010; Li et al., 2014; Björk et al., 2016; Zhong et al., 2016; Meng et al., 2017; Yang et al., 2018; Yang et al., 2019). We investigated HSF2 expression in various types of cancers and their corresponding normal tissues according to the TCGA database and observed that HSF2 was differentially expressed in 14 types of cancer (Figure 1). When combining the data from TCGA and GTEx, HSF2 was dysregulated in up to 25 types of cancer (Figure 1). HSF2 has been reported to be expressed at high levels in patients with lung cancer and affects the growth and migration of lung cancer cells by regulating the expression of HSPs (Zhong et al., 2016). HSF2 is also dysregulated in breast cancer cells to modulate their proliferation and invasion (Li et al., 2014; Yang et al., 2018). In breast cancer cells, HSF2 has been identified to mediate transcription of the miR-183/-96/-182 cluster, which is highly expressed to promote tumorigenesis by directly regulating RAB21 expression (Li et al., 2014). Moreover, HSF2 mediates expression of the ALG3 enzyme, which subsequently promotes the growth and migration of breast cancer cells (Yang et al., 2018). ALG3 silencing significantly suppresses tumor growth and downregulates HSF2 expression, suggesting the presence of a feedback loop between these two genes (Yang et al., 2018). Additionally, previous studies have shown a higher level of HSF2 expression in HCC than in normal liver tissues (Yang et al., 2019). Mechanistically, HSF2 interacts with euchromatic histone lysine methyltransferase 2 (EHMT2) to suppress the expression of fructose-bisphosphatase 1 (FBP1) (Yang et al., 2019). Knockdown of FBP1 facilitates the HIF1 activation and upregulates the expression of glucose transporter 1 (GLUT1), lactate dehydrogenase A (LDHA), and hexokinase 2 (HK2) to increase aerobic glycolysis in HCC (Yang et al., 2019). These results reveal that HSF2 may act as an oncogene to promote the initiation and progression of HCC. In addition, in ESCC, miR-202 inhibits apoptotic cell death by directly targeting HSF2, which subsequently affects the expression of HSP70 (Meng et al., 2017). In contrast, HSF2 expression is clearly decreased in prostate cancer tissues (Björk et al., 2016). The reduced expression of HSF2 is associated with the metastasis of prostate cancer, indicating that HSF2 is a tumor suppressor in prostate cancer (Björk et al., 2016). Altogether, these previous studies suggest that HSF2 may function as an oncogenic or tumor-suppressing gene in different tumors.

In view of the pathological and clinical significance of HSF2 across different cancer types, we also investigated whether HSF2 could be used as a potential biomarker for the early diagnosis of human cancers. Therefore, we examined the relationship between HSF2 expression and OS, DSS, DFI, and PFI across different cancer types (Figure 3; Supplementary Figures S2–4). The results indicated that high expression of HSF2 was a risk factor and associated with poor OS, DSS, DFI, and PFI in some cancers but seemed to be protective in KIRC and PAAD. Moreover, a nomogram including HSF2 and clinicopathological characteristics was constructed and exhibited good predictive power for the OS of KIRP, ACC, and LIHC patients (Figure 4; Supplementary Figure S5). These observations, together with the clinicopathological features, illustrate that HSF2 is a newly identified multicancer-relevant gene with prognostic potential in cancer risk prediction and they support the possible effect of HSF2 on lymph node metastasis in COAD, ESCA, LIHC, LUSC, and STAD.

To further explore the molecular mechanism by which HSF2 affects oncogenesis, we performed KEGG and GSEA analyses. The results directly demonstrated the involvement of HSF2 in colorectal cancer, renal cell carcinoma, hepatocellular carcinoma, endometrial cancer, small cell lung cancer, chronic myeloid leukemia, and viral carcinogenesis (Figures 6, 7). Moreover, HSF2 was found to be associated with ubiquitin-mediated proteolysis based on the KEGG and GSEA results (Figures 6, 7). A recent study indicated that the degradation of p53 was inhibited in HSF2-depleted cells by regulating the expression of PSMD10, an oncogene that interacts with the ubiquitin ligase MDM2 (Lecomte et al., 2010). In addition to PSMD10, the expression of some proteasome subunits, including PSMD1, PSMD2, PSMC4, ubb, and ubc, was also downregulated in the absence of HSF2 (Lecomte et al., 2010). As proteasome inhibition is an important strategy for the treatments of cancers, targeting HSF2 may be a valuable tool to reduce chemoresistance to proteasome inhibition. More importantly, we found that HSF2 was associated with various oncogenesis-related pathways, such as the cell cycle, Hippo signaling pathway, mismatch repair, ErbB signaling pathway, and mTOR signaling pathway (Figures 6, 7). Consistent with our previous studies, the results of the present study strengthen the important role of HSF2 in neurodegenerative diseases, including spinocerebellar ataxia (Figures 6, 7). Our previous study demonstrated that HSF2 deficiency accelerated disease progression and shortened lifespan in a mouse model of Huntington’s disease, suggesting that HSF2 could be a potential therapeutic target for neurodegenerative diseases by regulating the expression of αB-crystallin (CRYAB) (Shinkawa et al., 2011). Our GSEA results further implied that HSF2 was closely associated with the histone acetyltransferase complex (Figure 6). A previous study has shown that HSF2 can interact with WDR5, a core component of the Set1/MLL H3K4 histone methyltransferase complex (Hayashida, 2015). Moreover, HSF2 modifies active histone markers in the CRYAB promoter, including H3K4me3, H3K14Ac, and H3K27Ac (Hayashida, 2015). In fact, in addition to HSPs, HSF2 also regulates other target genes associated with oncogenesis, such as c-Fos (Wilkerson et al., 2007). Bioinformatics analysis has demonstrated that HSF2 may be involved in the oncogenesis of thyroid carcinoma by mediating the expression of SERPINA1 and FOSB (Lu and Zhang, 2016).

Oncogenesis is a complicated process accompanied by increased proliferation, resistance to cell death, enhanced angiogenesis, escape from immune surveillance, and tumor microenvironment (TME). The TME has attracted wide attention in cancer immunotherapy and has been identified as a main contributor to cancer initiation and development. It is well known that immunosurveillance affects the prognosis of cancer patients and that tumors can evade immune responses and immunotherapy by taking advantage of immune checkpoint genes, such as PD-1, PD-L1, and CTLA-4 (Gong et al., 2018; Kruger et al., 2019). Recently, immunotherapy has been recognized as an effective new strategy for cancer treatment. Although immunotherapy has made breakthroughs in cancer treatment, it still faces many challenges, and only a limited proportion of cancer patients respond well to immunotherapy (Gong et al., 2018; Kruger et al., 2019). Therefore, the identification of new targets and biomarkers is the key to further improving the efficacy of immunotherapy. Tumor-infiltrating immune cells, including B cells, T cells, dendritic cells, macrophages, and neutrophils, are the major part of the TME. Notably, our GSEA and KEGG results suggested that HSF2 was also involved in many immunity-associated pathways (IL−17 signaling pathway and the adaptive immune system) and various microbial infections (hepatitis B, shigellosis, Yersinia infection, and herpes simplex virus 1 infection) (Figures 5, 6). A recently study showed that HSF2 was upregulated in ulcerative colitis and was negatively associated with colon inflammation in mice (Wang W. et al., 2020; Zhang F. et al., 2021). NLRP3 inflammasome activation and IL-1β secretion are greatly enhanced in HSF2−/− DSS model mice (Zhang et al., 2020). Consistently, overexpression of HSF2 significantly suppresses inflammation-related processes, indicating that HSF2 participates in inflammation. Moreover, the expression of HSF2 is obviously higher in the intestinal mucosa of UC patients (Miao et al., 2014). More importantly, serum HSF2 levels are positively correlated with the expression of IL-1β and TNF-α. Knockdown of HSF2 potentiates the production of IL-1β and TNF-α induced by LPS (Miao et al., 2014; Wang W. et al., 2020; Zhang et al., 2020; Santopolo et al., 2021; Zhang F. et al., 2021). Here, to further estimate the relationships between HSF2 and the TME, we first examined the correlation of HSF2 expression and the abundance of different infiltrating immune cells across different cancer types. HSF2 expression was significantly linked with the abundance of infiltrating CD4^+^ T cells in 12 types of cancer, CD8^+^ T cells in 16 types of cancer, B cells in 16 types of cancer, macrophages in 17 types of cancer, neutrophils in 18 types of cancer, and DCs in 18 types of cancer (Figure 8). In addition, HSF2 positively correlated with these six types of immune cells in KIRC, LIHC, PAAD, and PRAD but negatively correlated with them in SARC (Figure 8A). We also used the xCell algorithm to further estimate the relationship between HSF2 expression and the level of infiltrating immune cells and found that HSF2 expression was significantly correlated with infiltrating CD4^+^ T helper (Th) cells and macrophages in most cancer types (Figure 8B). Tumor-associated macrophages (TAMs) within the TME have attracted great interest in basic science regarding their roles in metastasis, angiogenesis, and immunosuppression in various cancers (Mills et al., 2016; DeNardo and Ruffell, 2019; Anderson et al., 2021). The infiltration of TAMs in and around the tumor nest is one of the most important hallmarks of the process of cancer development. Macrophages consist of at least two subgroups, including proinflammatory M1 macrophages, and antiinflammatory M2 macrophages (Mills et al., 2016; Duan and Luo, 2021). M1 macrophages are cancer resistant due to their intrinsic phagocytosis, high antigen presenting capacity, and antitumor inflammatory activity. M1 macrophages also produce reactive oxygen species (ROS) and cytokines and are correlated with a favorable prognosis in cancer patients. In contrast, M2 macrophages are endowed with a repertoire of tumor-promoting capabilities associated with immunosuppression, angiogenesis, and neovascularization. A better understanding of their polarization into a protumoral phenotype to regulate tumor growth, angiogenesis, metastasis, and immune evasion prompted us to investigate their clinical significance as biomarkers in diverse cancers (Mills et al., 2016; DeNardo and Ruffell, 2019; Anderson et al., 2021; Duan and Luo, 2021). Here, we observed that HSF2 was significantly and negatively associated with the abundance of infiltrating macrophages, including M1 and M2 phenotypes, in most tumors, and illustrating the complexity of the TME. Moreover, accumulating evidence suggests that Th cells are essential to the development of the immune response and are involved in the response to antitumor immunotherapy (Basu et al., 2021; Renaude et al., 2021). T helper 1 (Th1) and T helper 2 (Th2) cells are the two predominant subtypes of CD4^+^ Th cells. Th1 cells play an antitumor role by orchestrating immunity against tumor cells. Th1 cells enhance the generation and function of CD8^+^ T cells, prevent angiogenesis, promote the senescence of tumor cells, and protect effector cytotoxic T lymphocytes from exhaustion; thus, modulating the Th1 cell response may lead to effective immune-based therapy (Basu et al., 2021; Laba et al., 2021; Renaude et al., 2021). Following differentiation, Th2 cells can produce IL-4, IL-5, IL-10, IL-13, and IL-17, not all of which are beneficial in cancer and contribute to tumor growth and metastasis. Simultaneously, infiltration of Th2 cells in the TME is usually connected with a poor prognosis in human cancers (Basu et al., 2021; Laba et al., 2021; Renaude et al., 2021). In the present study, we found that HSF2 expression was negatively associated with the infiltration level of Th1 cells but positively correlated with the level of infiltrating Th2 cells in most tumor types. More directly, we also investigated the effects of HSF2 expression on immune modulators, including immunoinhibitors, immunostimulators, chemokines, and MHC-related genes (Figure 9). Intriguingly, we observed that the expression of HSF2 was greatly associated with most immunoinhibitors, including PD-1, PD-L1, and CTLA4, in OV, PAAD, PRAD, and LIHC (Figure 9). Furthermore, HSF2 was also significantly correlated with the expression of different chemokines in PAAD, PRAD, and SARC; positively associated with immunostimulators in HNSC, KICH, KIRC, LIHC, OV, PAAD, and PRAD; and strongly correlated with most MHC-related genes in KIRC, OV, PAAD, PRAD, LGG, and SARC (Figure 9). Additionally, HSF2 expression significantly affected the expression of well-known immune checkpoints (Figure 11). TMB influences the possibility of generating immunogenic peptides, therefore affecting the response to immunotherapy in cancer patients. MSI is another important index for predicting oncogenesis and tumor development. Therefore, TMB and MSI could act as predictive factors for the efficacy of immune checkpoint inhibitors (Sha et al., 2020; Li et al., 2021). Here, we observed that the expression of HSF2 was associated with TMB and MSI in several cancer types (Figure 10). These results provide further clues regarding the correlation between HSF2 expression and cancer immunity. Based on our observations, targeting HSF2 may be a promising immunotherapeutic strategy for the treatment of specific cancers. Until now, there have been no small molecule drugs specifically targeting HSF2. Efforts are needed to develop novel drugs or RNAi techniques targeting HSF2 in tumor-infiltrative immune cells. Conversely, engineering tumor-specific macrophages and Th cells by modulating HSF2 expression may also be a promising strategy to increase the efficacy of immunotherapy.

Our results may provide better prognostic prediction and immune-oncological perspectives regarding the application of HSF2 as a prognostic biomarker. However, despite performing these bioinformatics analyses by collecting information from various databases, this study has several limitations. First, some contradictory findings of individual cancers in different databases were observed. It is therefore necessary to further investigate the expression and function of HSF2 using a large sample size. A deeper understanding of these differences may facilitate the development a global view to generate cancer development mechanisms with HSF2 expression. Second, although the signaling pathways and prognostic value of HSF2 in different cancer types were explored, there were no *in vitro* or *in vivo* experiments to verify these findings. Third, the effects of HSF2 on immune cell infiltration and immunotherapy in human cancer require experimental and clinical validation.

## Conclusion

The results of the current study reveal the varied expression of HSF2 in different types and stages of cancers, which suggests that the effects of HSF2 on oncogenesis may vary across different cancer types. A significant correlation between HSF2 expression and the prognosis of cancer patients was observed. HSF2 expression was strongly related to immune cell infiltration, immune checkpoint genes, TMB, and MSI. The present study integrated existing data to explore the potential function of HSF2 in cancers and provides insights for targeting HSF2 to improve the therapeutic efficacy of immunotherapy.

## Data Availability

The original contributions presented in the study are included in the article/Supplementary Material, further inquiries can be directed to the corresponding author.
