# Supplementary figures and images for: Pan-Cancer Integrated Analysis of HSF2 Expression, Prognostic Value and Potential Implications for Cancer Immunity
Source: Front Mol Biosci. 2022 Jan 11;8:789703. doi: 10.3389/fmolb.2021.789703 (PMC8787226; doi:10.3389/fmolb.2021.789703)

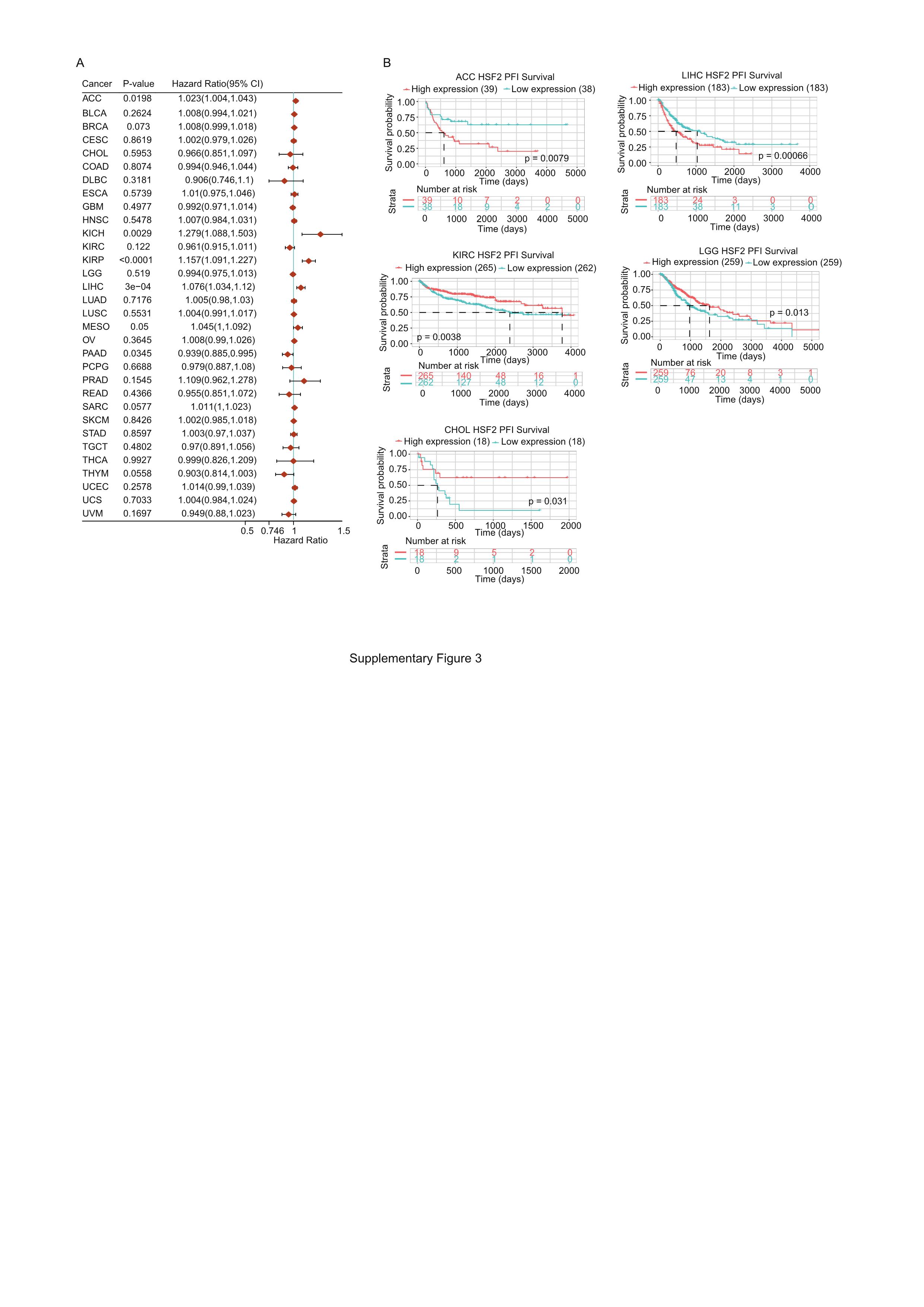

Supplement: Supplementary file 1 [file Image3.JPEG]

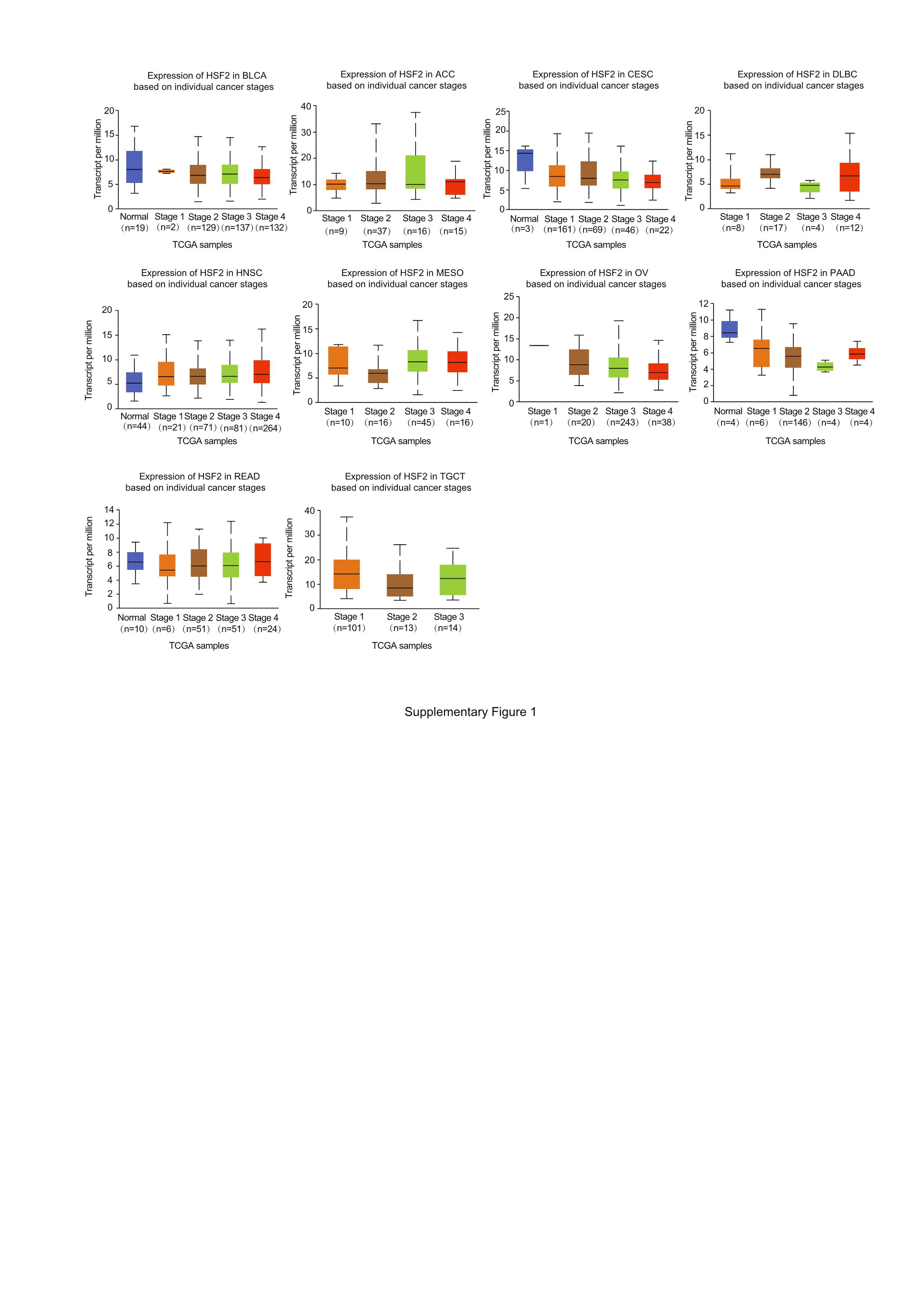

Supplement: Supplementary file 2 [file Image1.JPEG]

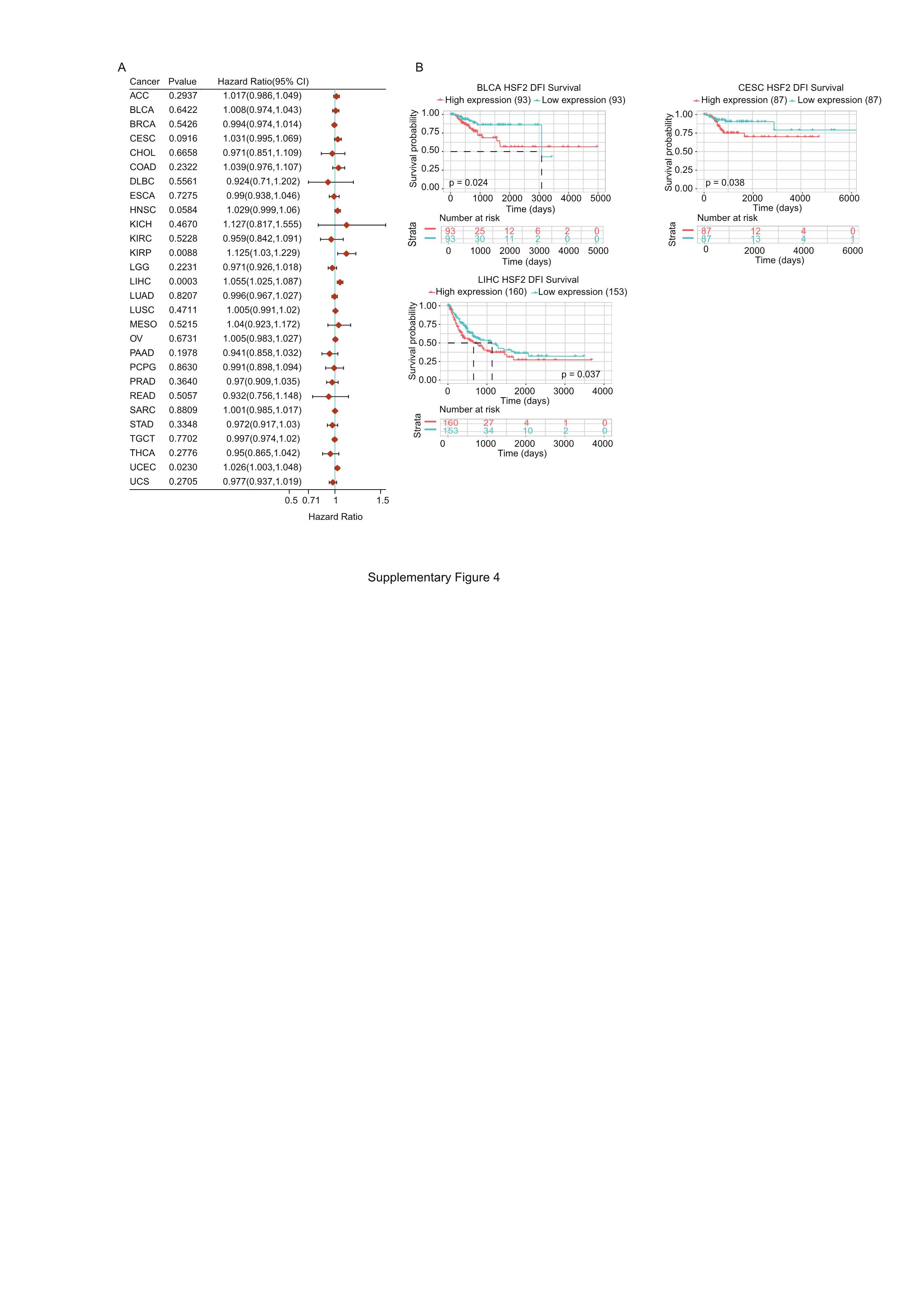

Supplement: Supplementary file 3 [file Image4.JPEG]

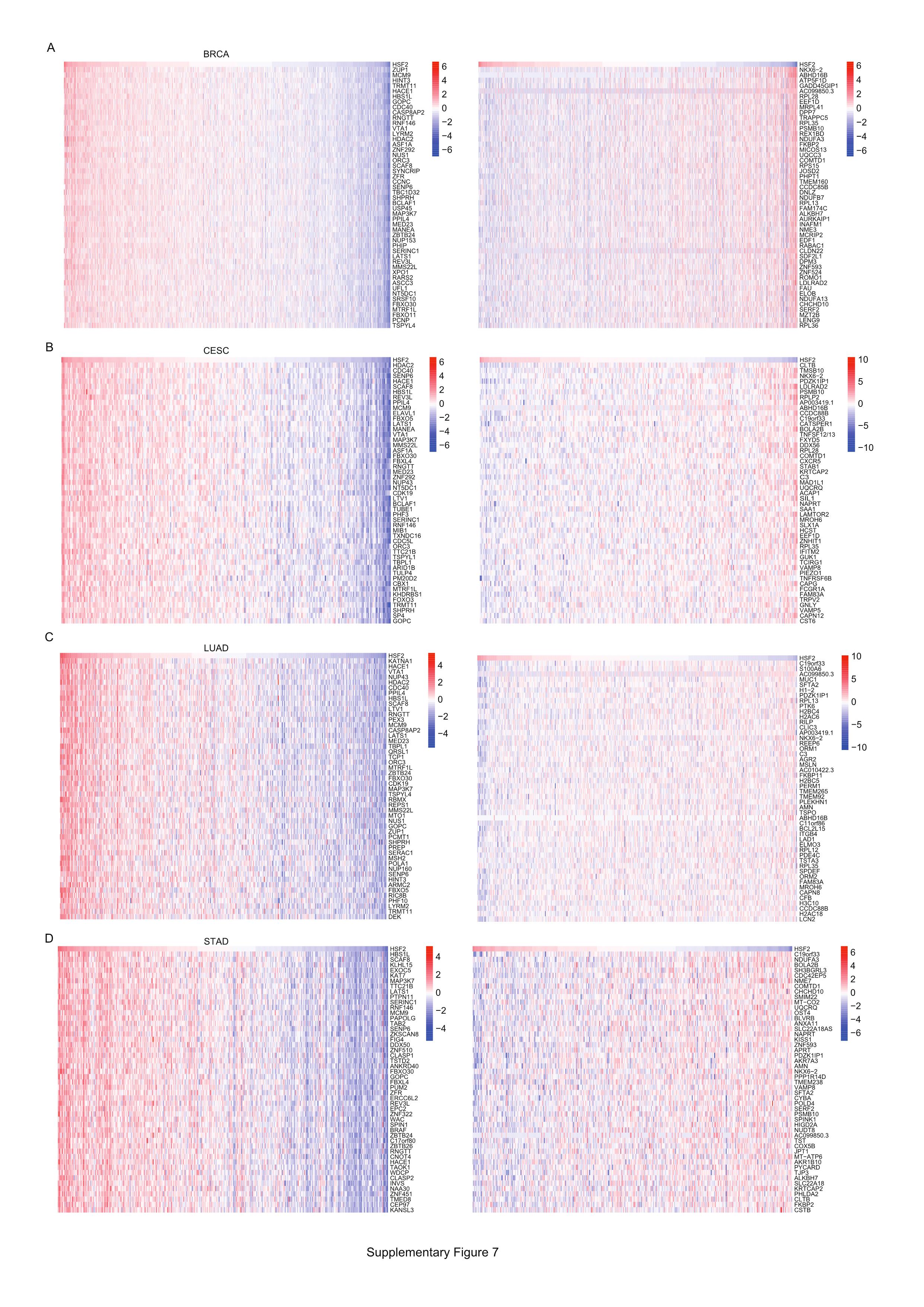

Supplement: Supplementary file 4 [file Image7.JPEG]

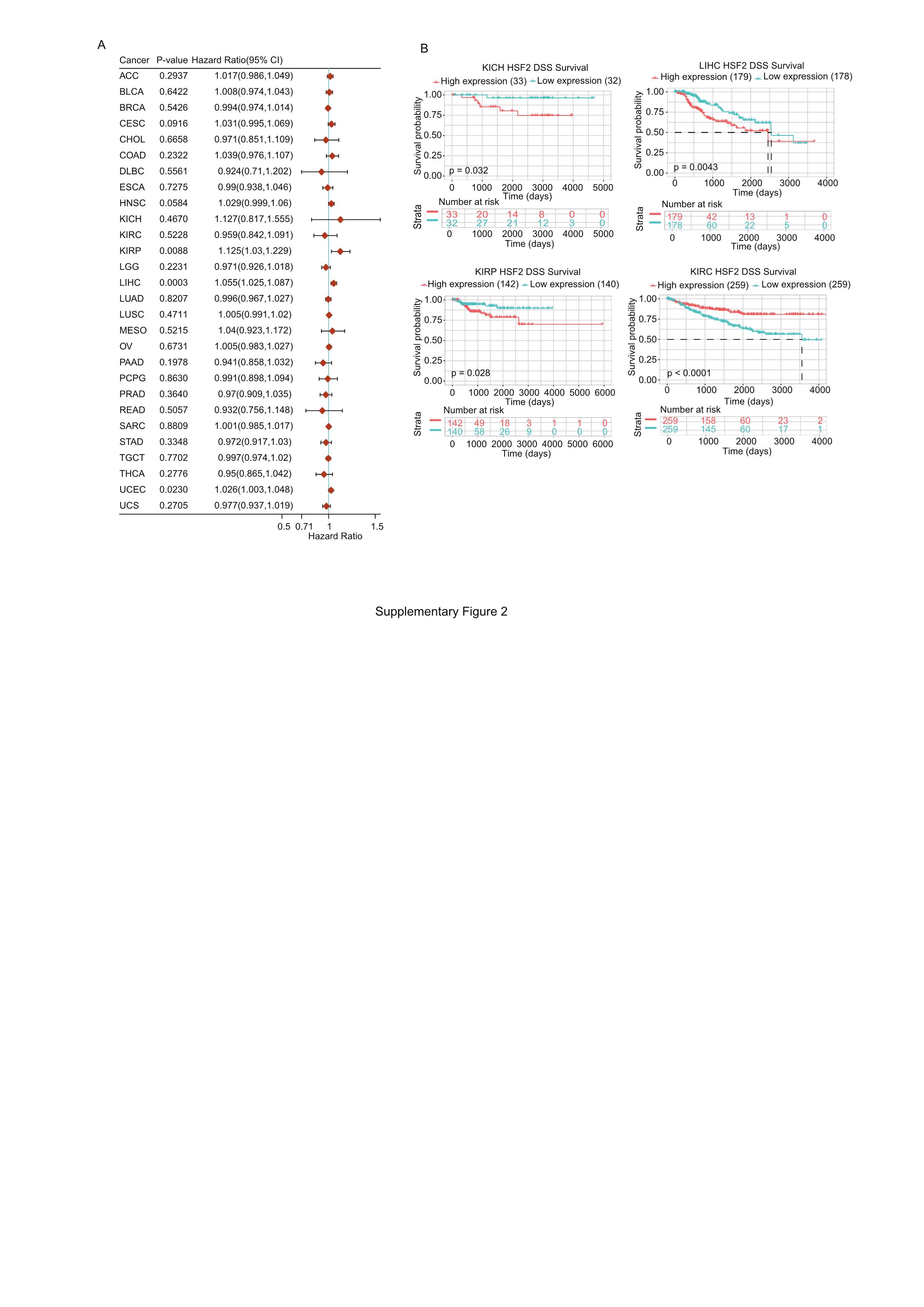

Supplement: Supplementary file 5 [file Image2.JPEG]

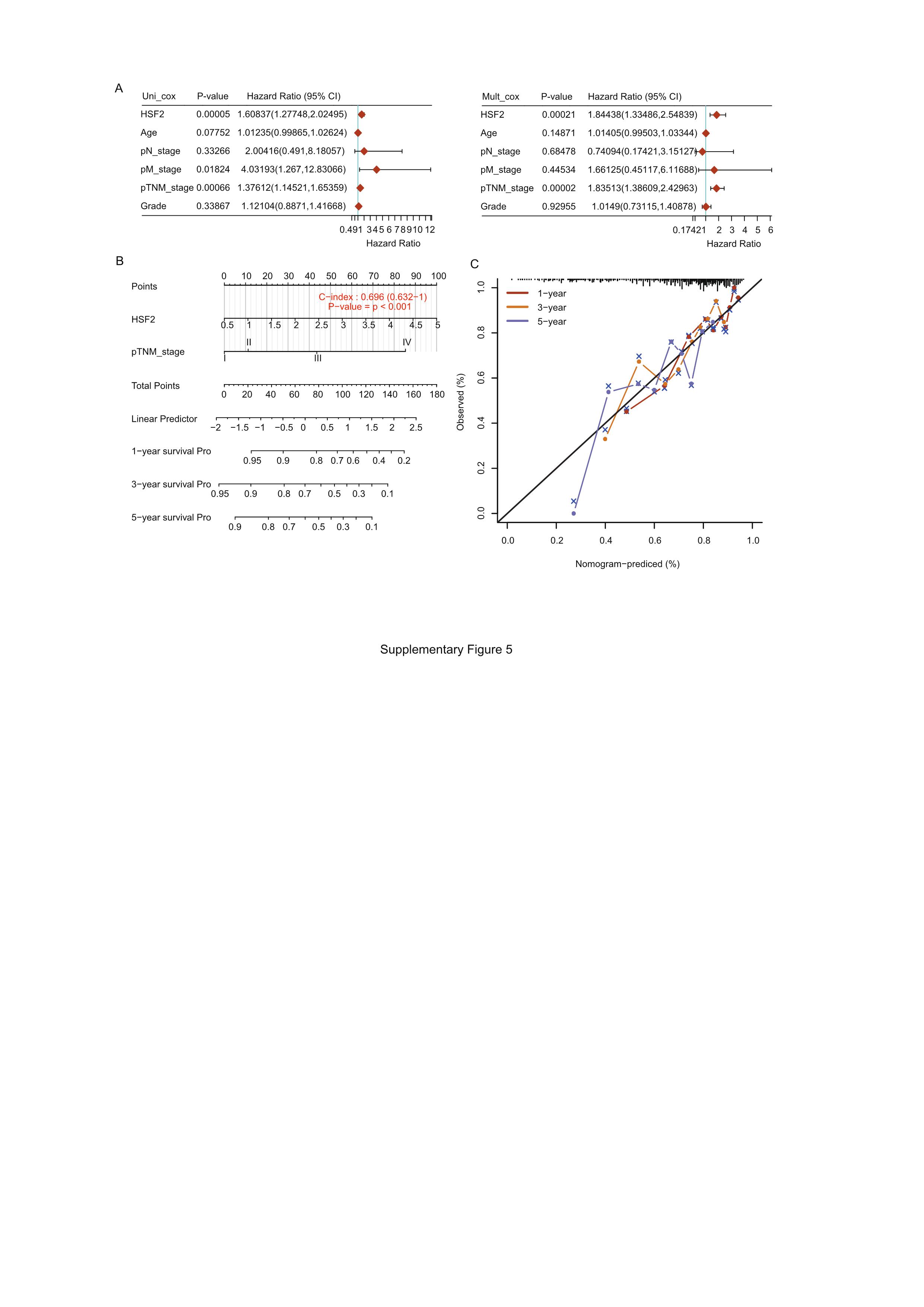

Supplement: Supplementary file 6 [file Image5.JPEG]

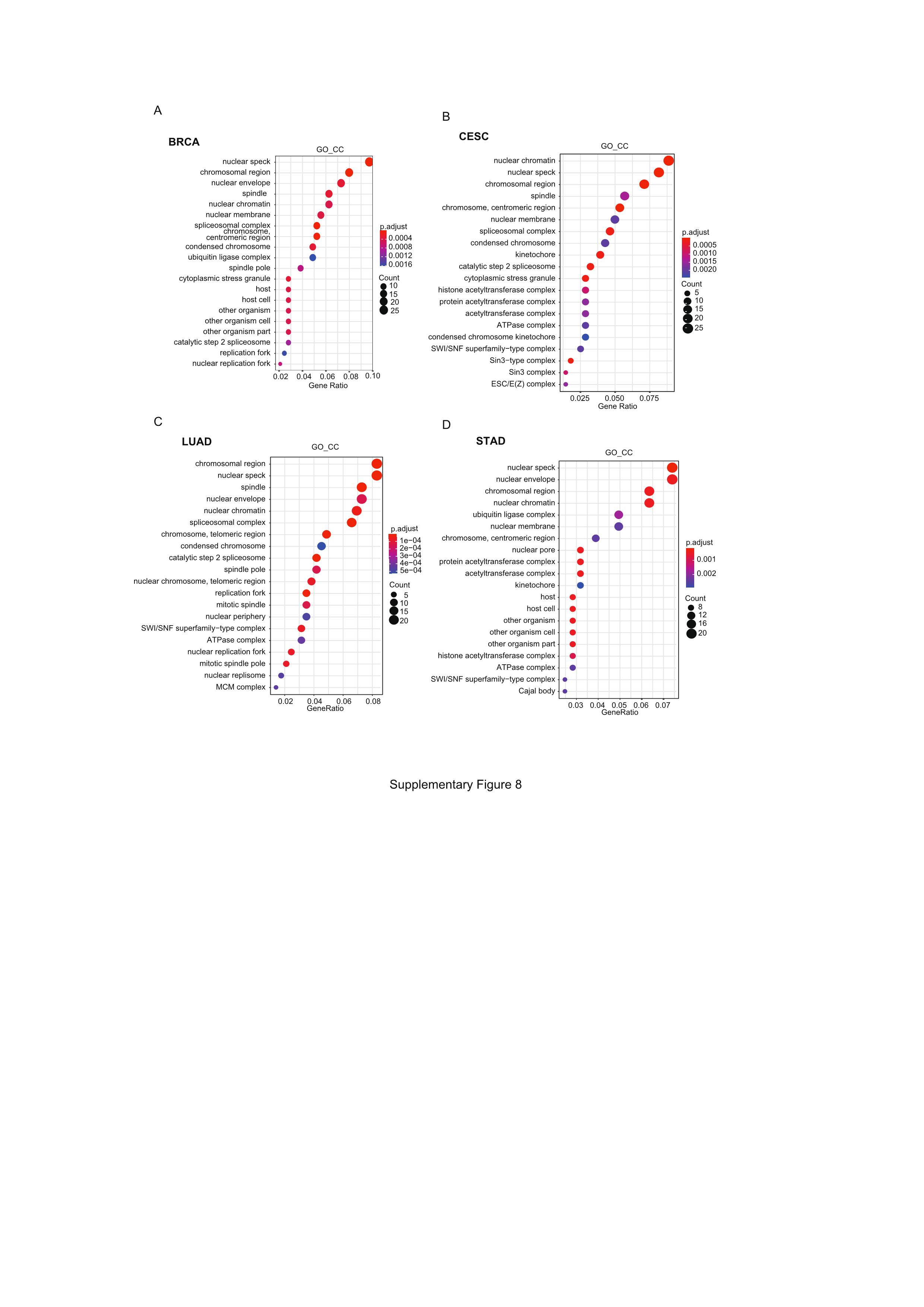

Supplement: Supplementary file 7 [file Image8.JPEG]

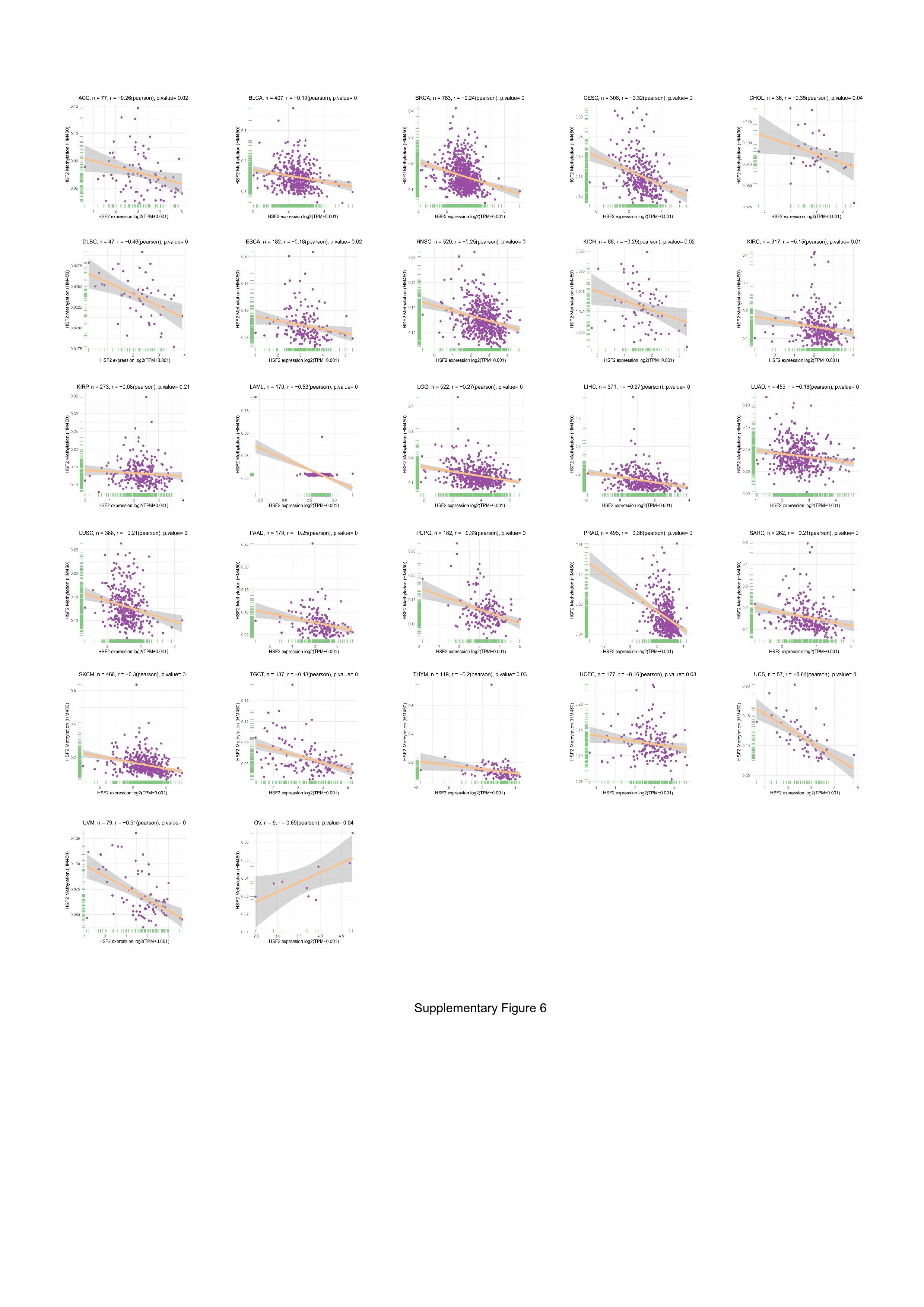

Supplement: Supplementary file 8 [file Image6.JPEG]
